# Research and Development of High-Performance High-Damping Rubber Materials for High-Damping Rubber Isolation Bearings: A Review

**DOI:** 10.3390/polym14122427

**Published:** 2022-06-15

**Authors:** Bowen Chen, Junwu Dai, Tingsu Song, Qingsong Guan

**Affiliations:** 1 Key Laboratory of Earthquake Engineering and Engineering Vibration, Institute of Engineering Mechanics, China Earthquake Administration, Harbin 150086, China; junwu@iem.ac.cn; 2 Key Laboratory of Earthquake Disaster Mitigation, Ministry of Emergency Management, Harbin 150086, China; 3 Shenzhen Academy of Disaster Prevention and Reduction, Shenzhen 518003, China; saviour_sts@163.com; 4 Quakesafe Technologies Co., Ltd., Kunming 650100, China; qsguandream@126.com

**Keywords:** high-damping rubber isolation bearings, high-damping rubber materials, effective damping temperature range, damping loss factor, temperature dependence

## Abstract

At present, high-damping rubber materials, widely used in the field of engineering seismic isolation, generally have the problems such as narrow effective damping temperature range, low damping loss factor and strong temperature dependence, which lead to prominent dependence of temperature and load conditions of the isolation performance of high-damping rubber isolation bearings. Research and development of high-performance high-damping rubber materials with broad effective damping temperature range, high damping loss factor and weak temperature dependence are very urgent and necessary to ensure the safety of the seismic isolation of engineering structures. This paper mainly reviews the recent progress in the research and development of high-damping rubber materials using nitrile butadiene rubber (NBR), epoxidized natural rubber (ENR), ethylene propylene diene rubber (EPDM), butyl rubber (IIR), chlorinated butyl rubber (CIIR), and bromine butyl rubber (BIIR). This is followed by a review of vulcanization and filler reinforcement systems for the improvement of damping and mechanical properties of high-damping rubber materials. Finally, it further reviews the constitutive models describing the hyperelasticity and viscoelasticity of rubber materials. In view of this focus, four key issues are highlighted for the development of high-performance high-damping rubber materials used for high-damping rubber isolation bearings.

## 1. Introduction

Seismic isolation technology is an effective method to weaken the destructive effects of earthquakes by reducing the base shear and accelerations transmitted to the superstructure during the earthquake [1]. It has been widely used in earthquake-prone countries such as Japan, China, New Zealand, Italy, and the United States [2]. High-damping rubber isolation bearing is one of the most popular seismic isolation devices used for engineering seismic isolation [3]. The materials used for the production of high-damping rubber bearings mainly consist of the high-damping rubber and the steel plate, among which the properties of high-damping rubber materials have a direct impact on the anti-seismic performance of high-damping rubber isolation bearings. However, at present, high-damping rubber materials with inferior damping properties such as narrow effective damping temperature range, low damping loss factor and strong temperature dependence will lead to a low stability of isolation performance of high-damping rubber isolation bearings due to the strong dependence of the temperature and load conditions. Therefore, it is very urgent and necessary to develop high-performance high-damping rubber materials with a broad effective damping temperature range, high damping loss factor and weak temperature dependence to ensure the safety of seismic isolation of engineering structures.

First, this paper briefly introduces the seismic isolation technology and high-damping rubber isolation bearings. In the main text, recent progress in the research and development of high-damping rubber materials using nitrile butadiene rubber (NBR), epoxidized natural rubber (ENR), ethylene propylene diene rubber (EPDM), butyl rubber (IIR), chlorinated butyl rubber (CIIR), and bromine butyl rubber (BIIR) are reviewed, in that order. This is followed by a review of vulcanization and filler reinforcement systems for the improvement of damping and mechanical properties of high-damping rubber materials. Finally, it further reviews the constitutive models describing the hyperelastic and viscoelastic behaviors of rubber materials. In view of this focus, four key issues are highlighted for the development of high-performance high-damping rubber materials used for high-damping rubber isolation bearings.

## 2. Seismic Isolation Technology

As an unexpected natural disaster, earthquakes are highly unpredictable and destructive. The damage of artificial structures during the earthquake is the direct cause of casualties and property losses [4]. The development of anti-seismic technology helps to protect engineering structures from the earthquake damage. Traditional anti-seismic technology harnessed by engineering structures mainly relies on the hysteretic energy dissipation of structures to realize the dissipation of seismic energy. However, these structures dissipate the seismic energy by their own elastoplastic deformation, which would eventually causes the damage of themselves. Furthermore, the more seismic energy is dissipated, the greater the degree of damage. Thus, traditional anti-seismic technology is unable to meet the anti-seismic design requirements of engineering structures.

**Figure 1:**
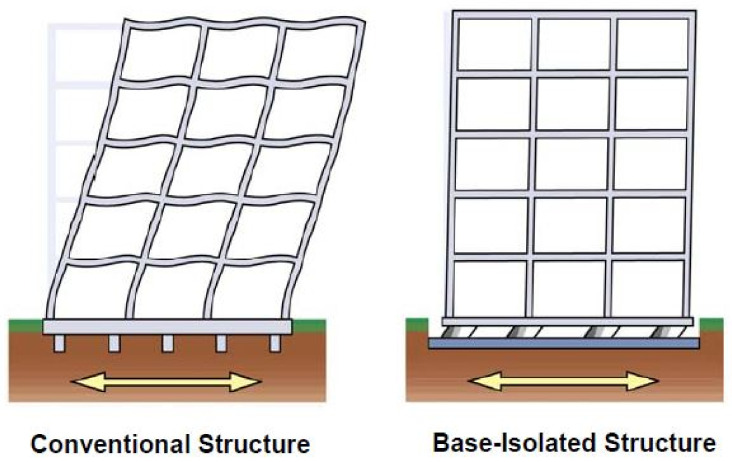
Schematic representation of deformations of conventional non-isolated and base-isolated structures [5].

**Figure 2:**
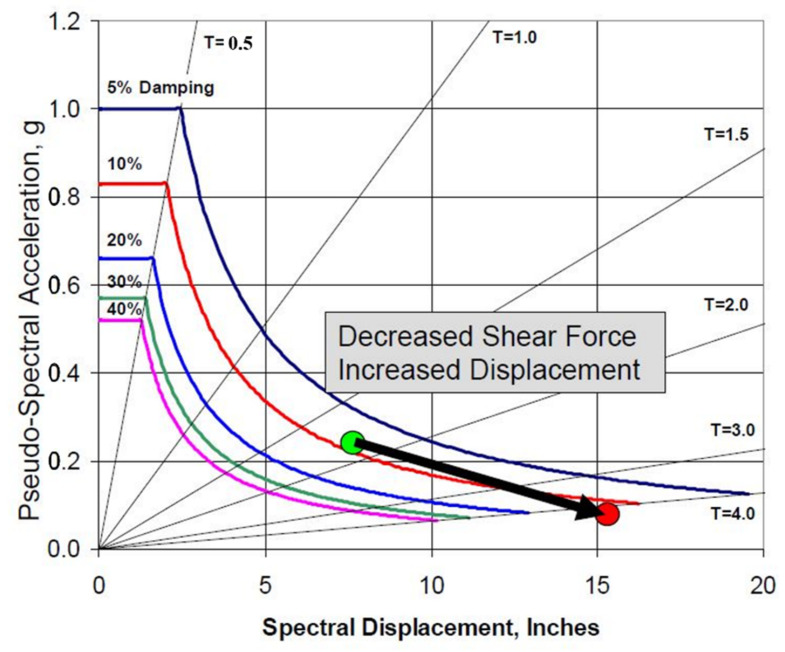
Effect of seismic isolation on the structure from the perspective of Acceleration Displacement Response Spectra (ADRS) [5].

In recent decades, reinforcement and reconstruction of structures have been in great demand with the increasing public concern about the anti-seismic fortification. Seismic isolation technology of structures exhibits remarkable anti-seismic capabilities and cost savings because of its great compatibility with the reinforcement of structures. In contrast with traditional anti-seismic technology, the seismic isolation technology is used to isolate structures from the ground by the installation of a seismic isolation layer, which is composed of flexible seismic isolation devices at the bottom or between a certain layer of a structure. It is an anti-seismic technology to protect the structure from the earthquake damage by consuming and buffering the vibration of the superstructure caused by the ground during the earthquake through the deformation of the seismic isolation layer. The difference in the superstructure response of a conventional non-isolated structure and a base-isolated structure is qualitatively illustrated by Figure 1. During an earthquake, base-isolated structures experience deformations within the isolation devices and the acceleration responses are relatively uniform over the height of the superstructure. In contrast, conventional non-isolated structures experience inter-story drifts and amplified accelerations at upper floor levels [5]. As shown in Figure 2, in ADRS spectrum, the natural period of the superstructure with a certain value of damping ratio increases when an isolation system is installed, which results in an increase in the spectral displacement and a decrease in the pseudo-spectral acceleration, as indicated by the arrow from the green circle to the red circle. When the natural period of the superstructure stays constant, the spectral displacement decreases with the increase in the damping ratio. In addition, since the seismic isolation technology dissipates the seismic energy such that the superstructure can be designed with a lower level of seismic fortification intensity, which significantly reduces the consumption of building materials such as steel and cement, especially in areas with high seismic fortification intensity, the effective control and the reduction of emission of greenhouse gases is achieved.

## 3. High-Damping Rubber Isolation Bearing

Among seismic isolation devices, rubber isolation bearing is currently most widely used. It is a mature isolation technology, which includes the ordinary natural rubber isolation bearing, the lead-core rubber isolation bearing and the high-damping rubber isolation bearing. The rubber isolation bearing consists of alternating rubber layers bonded between thin steel shims, which is vulcanized at high temperature and pressure [6,7]. It could ensure the bearing capacity and the vertical stiffness to constantly and stably support the weight of the building. Furthermore, the horizontal stiffness of the building could be greatly reduced, which produces a large horizontal deformation to adapt to the relative horizontal displacement between the building and the base [8]. The high elasticity of rubber materials enables the rubber isolation bearings to absorb and dissipate seismic energy during the earthquake and to have a remarkable recentering ability. In [9], completed test results have shown that rubber isolation bearings shift the natural period of a structure to the long period range and reduce the response of its superstructure. Therefore, rubber isolation bearing is a seismic isolation technology with a widespread prospect of applications.

**Figure 3:**
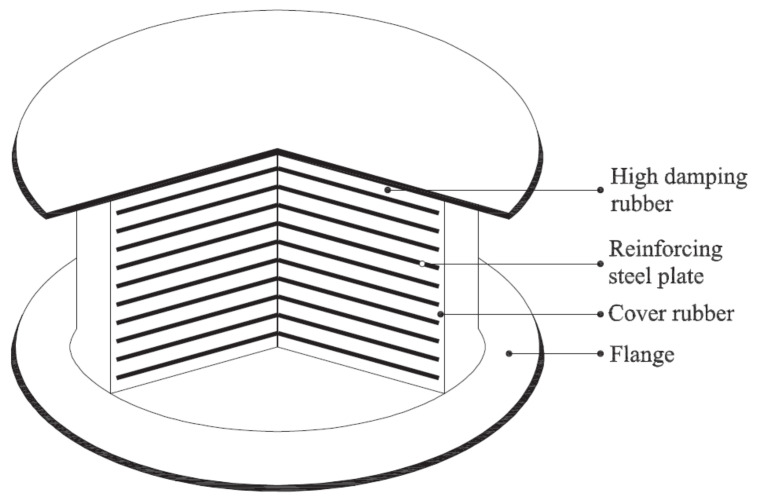
Cross-section of high-damping rubber isolation bearing [13].

**Figure 4:**
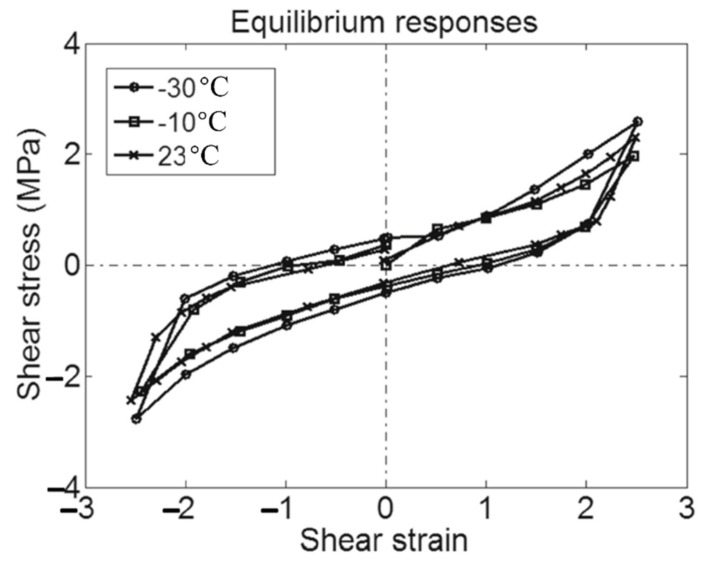
Equilibrium responses of a high-damping rubber isolation bearing obtained from multi-step relaxation (MSR) tests at temperatures of −30, −10 and 23 °C [21].

In recent years, rubber isolation bearings have undergone new changes and developments. Considerable efforts have been invested in the development of new rubber isolation bearings [10,11,12,13,14,15,16,17,18]. Compared to ordinary natural rubber isolation bearings and lead-core rubber isolation bearings, high-damping rubber isolation bearings (Figure 3) have plenty of advantages over the both, such as a simple structure, stable mechanical performance, strong energy dissipation capacity, large stiffness before yielding, environmental protection, etc., that make it an excellent choice for base-isolated structures [16]. Substantial manpower and material resources have been invested in the development of high-performance high-damping rubber materials for high-damping rubber isolation bearings. Using the rubber modification technology, the damping performance of high-damping rubber materials is far superior than that of pure natural rubbers, such that high-damping rubber isolation bearings could obtain a higher equivalent damping ratio of more than 20%, thereby achieving the enhancement of the seismic isolation effect. Furthermore, replacing the lead-core by high-damping rubber materials avoids the leakage of lead into the surroundings as soon as the bearing is damaged, which is applicable for those regions with special requirements for environmental protection. However, the issue of performance stability of high-damping rubber isolation bearings is always a bottleneck, which hinders the practical application of high-damping rubber bearings. The influence of temperature on the performance of rubber isolation bearings mainly includes: (i) the instantaneous change in rubber materials at ambient temperature; (ii) the crystallization hardening of the rubber induced by long-term exposure in the low-temperature environment; (iii) the increase in the internal temperature of rubber isolation bearings caused by the hysteretic energy dissipation and iv) the effect of seasonal changes in temperature on the life span of rubber isolation bearings [19]. In [20], property evolutions of a high-damping rubber isolation bearing were investigated within the temperature range of −20–40 °C, in which the tests were carried out under a vertical compressive stress of 12 MPa and a horizontal shear strain of 100%. It was found that the hysteretic energy dissipation, the yield strength and the equivalent damping ratio of high-damping rubber isolation bearings decrease significantly with the increase in the temperature. Meanwhile, the aforementioned properties were obtained before and after the aging test of the bearing under a high-temperature aging condition of 100 °C for a period of 14 days. The comparison results showed that the high-temperature aging of the bearing degraded its mechanical properties by 2–8%. In [21], simple shear, simple and multi-step stress relaxation (shear deformation under a constant vertical compressive average stress of 6 MPa) tests were performed on high-damping rubber isolation bearings at different temperatures of −30, −10 and 23 °C. The low-temperature dependence of mechanical properties and the temperature dependence of viscosity of high-damping rubber isolation bearings were, respectively, investigated. The results of the simple shear tests showed that the area of hysteresis loop of the rubber bearing increased with the decrease in the temperature. When the high-damping rubber isolation bearings were sheared to different strains of 100%, 150% and 175%, the results of simple stress relaxation tests showed a strong temperature dependence of viscosity of the high-damping rubber isolation bearing, leading to a high rate sensitivity of the bearing at low temperatures. Furthermore, a weak effect of low temperatures on the equilibrium stress was observed after the bearing was relaxed. Figure 4 illustrates the equilibrium responses of a high-damping rubber isolation bearing obtained from multi-step relaxation (MSR) tests at different temperatures of −30 °C, −10 °C and 23 °C. It could be observed from Figure 4 that the area of hysteresis loop significantly changes with the increase in the temperature.

From this point of view, high-damping rubber materials need to maintain their high-damping performance at different ambient temperatures and large shear strains (≥200%), due to the significant difference in climatic conditions in various regions. Moreover, with the increase in the vibration frequency and amplitude, hysteretic heat generation of the rubber itself will significantly affect the damping properties of high-damping rubber materials, thereby restricting high-damping rubber isolation bearings to exert their isolation performance and limiting the scope of their practical applications [22].

With the expansion of application demand and scope of high-damping rubber isolation bearings and further design requirements for seismic isolation structures, high-damping rubber materials, at present, cannot meet the design standards of high-damping rubber isolation bearings. Therefore, the development of new high-damping rubber materials with a broad effective damping temperature range, high damping properties and weak temperature dependence is the key solution to design high-damping rubber isolation bearings. Furthermore, the research and development of high-performance high-damping rubber materials with high strength, high elasticity, high flexibility, high damping properties and their weak temperature dependence is the future trend.

## 4. High-Damping Rubber Materials

### 4.1. Damping Properties

Rubber materials belong to high polymer damping materials with the nature of hyperelasticity and viscoelasticity [23]. When the material is subjected to dynamic external force, the strain induced will lag behind the stress variation. The loss of mechanical energy generated in this process is reflected by the damping effect of rubber materials. The damping performance of rubber materials is mostly characterized by the damping loss factor (tan δ). The larger the damping loss factor, the higher the damping energy dissipation of the material, which can be described by the expression $\tan\delta =E^{\prime \prime }/E^{\prime }$, where δ is the phase angle (mechanical loss angle) at which the strain lags behind the stress; $E^{\prime }$ is the storage modulus, which characterizes the energy storage in the material under the action of stress; $E^{\prime \prime }\;$ is the loss modulus, which characterizes the energy loss.

The damping properties of a high-damping rubber material include the effective damping temperature range, the damping loss factor and its temperature dependence in the effective damping temperature range. International Standard Technical Committee ISO/TC45 requires that the damping loss factor of rubber materials used for seismic isolation bearings should be greater than 0.1 in the frequency range of 0.2–5 Hz and in the strain range of 10–200%. Moreover, rubber materials for outdoor surroundings should be guaranteed to have a damping loss factor greater than 0.3, at least in the temperature range from 60 to 80 °C [24,25]. However, most of rubber materials have an effective damping temperature range of 20–40 °C, which lies below room temperature [25]. The synthesis of rubber materials used for manufacturing high-damping rubber isolation bearings should simultaneously consider the damping and mechanical properties of the rubber material. According to the Chinese Standard JG/T-118-2018 [26], the brittleness temperature of high-damping rubber materials used for high-damping rubber isolation bearings should be below −40 °C, the tensile strength and the elongation at break should be above 10 MPa and 550%, respectively, and the compression set at 70 °C × 24 h should be less than 60%.

In [22], a Double-Target model was used to investigate the effect of property evolutions of high-damping rubber materials on the seismic response of base-isolated structures. Numerical simulation results showed that dissipated energy of high-damping rubber materials caused temperature rises of 13, 15 and 20 °C under both the surrounding temperatures of −10 and 20 °C, respectively. Results showed that the variations in damping capacity and rigidity of high-damping rubber materials with the temperature will significantly affect the seismic response of the base-isolated structures. The maximum displacement difference and the maximum inter-story displacement difference of structural responses can reach 10% and 20%, respectively. In [27], uniaxial monotonic tensile tests (strain range of 0–80%, strain rate of 2 × 10^−1^ s^−1^, 2 × 10^−2^ s^−1^, 2 × 10^−3^ s^−1^ and 2 × 10^−4^ s^−1^) and cyclic tensile tests (strain range of −20–80%, strain rate of ±2 × 10^−1^ s^−1^) were conducted on carbon black filled rubber materials at −20, 23, 60 and 100 °C, respectively. Results showed that the area of hysteresis loop of the rubber material decreases with the increase in the temperature. The material has a higher rate sensitivity at a low temperature of −20 °C, and the equilibrium stress curve of the rubber material at a high temperature of 100 °C had a larger deviation from those of the other temperatures. Above all, the improvement of damping properties of high-damping rubber materials under the premise of meeting design requirements is the key to the development of high-damping rubber materials.

The damping properties of rubber materials are highly dependent on the material composition and the thermochemical processing [28]. At present, rubber blending and interpenetrating polymer network (IPN) technologies are two relatively mature technical tools, to broaden the effective damping temperature range of rubber materials [29]. Though IPN technology can be used to synergistically combine polymer materials with different properties and form new polymer materials with high mechanical properties [30], the rubber synthesis process using IPN technology is complex and cost-intensive, which greatly limits the application of IPN polymers, as structural damping materials, in the production of high-damping rubber isolation bearings [31].

### 4.2. Rubber Blending Technology

**Figure 5:**
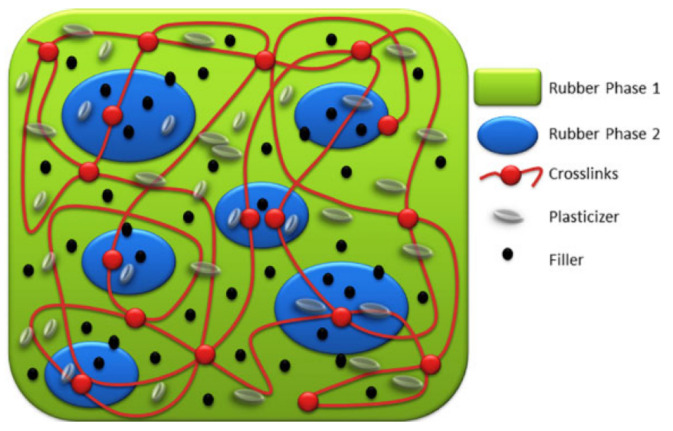
Pictorial representation of a cross-linked typical rubber blend with additives [33].

**Figure 6:**
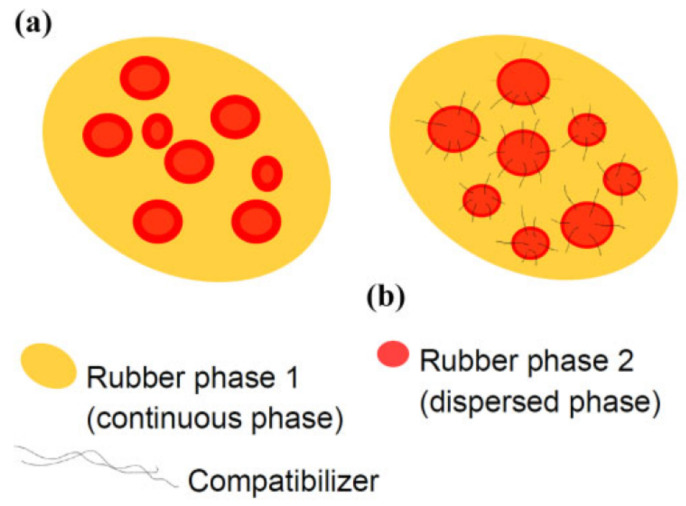
Schematic representation of (**a**) incompatible rubber-rubber blends, and (**b**) compatible rubber-rubber blends [33].

Rubber blending technology is a traditional rubber modification method that can better improve the properties of monomer rubber materials [32]. In recent years, researchers have achieved many excellent results using rubber blending technology to develop high-damping rubber materials for high-damping rubber isolation bearings. Generally, rubber components blended in the rubber matrix materials include natural rubber (NR), nitrile butadiene rubber (NBR), epoxidized natural rubber (ENR), ethylene propylene diene rubber (EPDM), butyl rubber (IIR), chlorinated butyl rubber (CIIR), and bromine butyl rubber (BIIR). The morphological features of rubber blends, to a large extent, determine the properties of rubber blends [33]. Control of this morphology, along with partitioning of compounding additives such as vulcanizing agents and fillers between different rubber phases, provide opportunities for achieving desired properties [34]. Figure 5 shows a pictorial representation of an even distribution of carbon black fillers between two rubber phases. In addition, the compatibility between rubber phases plays a crucial role in the performance of rubber blends. Figure 6 shows the schematic representations of compatible and incompatible rubber blends, respectively. Poor compatibility mostly causes phase separation of rubber blends, especially at elevated temperatures, which would have an impact on the life span of rubber material products in service. Apart from experiments, molecular dynamics simulation is another valuable tool in the evaluation of the compatibility and the prediction of phase behaviors of polymer blends, particularly in case the polymers under consideration are not available [35].

**Figure 7:**
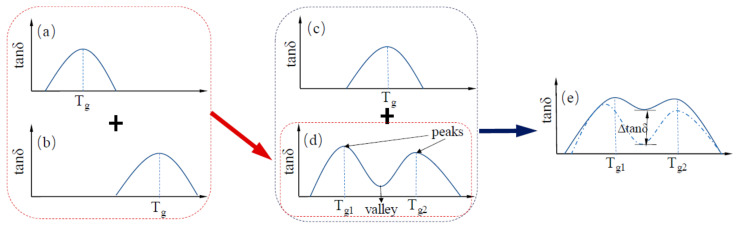
Schematic representation of the variation in the damping loss factor of rubber blends with respect to the temperature: (**a**–**c**) shows the dynamic mechanical analysis (DMA) temperature spectrum of each rubber component, respectively and (**d**,**e**) shows the broadening of the effective damping temperature range of the rubber blends [31].

Two rubbers with different polarities will form a so-called “sea island” structure in the rubber blend. Due to the difference in solubility, two damping peaks in the rubber blend will appear near their respective glass transition temperatures and a damping valley will be formed between them. However, the difference in solubility will affect the compatibility between rubbers, thereby reducing the stress transfer between the molecules of two rubber phases causing the phase separation of the rubber blend under the action of external forces, which greatly affects the performance and service life of the rubber material [36]. Therefore, to improve the compatibility of two rubber components, a third polymer component with its solubility in between the both is mostly introduced as a compatibilizer of these two rubber components. When a third rubber component is mixed, the damping valley is uplifted, thereby broadening the effective damping temperature range of the rubber. This process can be depicted by Figure 7.

### 4.3. NBR Composites

Nitrile butadiene rubber (NBR) is a high polymer elastomer obtained by emulsion or solution polymerization of butadiene and acrylonitrile monomers. High compatibility and strong intermolecular interaction between NBR and hindered phenol (AO-80) enable NBR/AO-80 rubber composites to produce strain-induced orientation in the deformation state [37]. In [38], NBR/AO-80 rubber composites with blend ratios of 100/20, 100/40, 100/60, and 100/100 were prepared, and the morphology, the damping and mechanical properties of the material were obtained. Scanning electron microscope (SEM) and dynamic mechanical thermal analysis (DMTA) (temperature range of −60–150 °C, frequency of 1 Hz, heating rate of 3 °C/min) showed that the addition of AO-80 small molecules produced an intermolecular hydrogen bond between NBR and AO-80. With the increase in AO-80 content, the damping peak value increased from 1.95 to 3.19, which was accompanied by broadening the temperature range near the damping peak. The glass transition temperature of the material moved towards high temperatures, noticeably from −21 to 23 °C, while the material has an effective damping temperature range (temperature range where the damping loss factor is greater than 0.3) of 42 °C, which did not meet the requirements of the effective damping temperature range of 60–80 °C of rubber materials for outdoor surroundings. The tensile test results showed that the mechanical properties were improved with the increase in loadings of AO-80, as shown in Table 1. The hardness (Shore A) and the permanent set of NBR/AO-80 rubber composites reached, respectively, their maximums of 88 and 8% when the loadings of AO-80 reached 100 phr. The tensile strength and the elongation at break reached their maximums of 17.3 MPa and 703%, respectively, when the loading of AO-80 was 60 phr.

**Figure 8:**
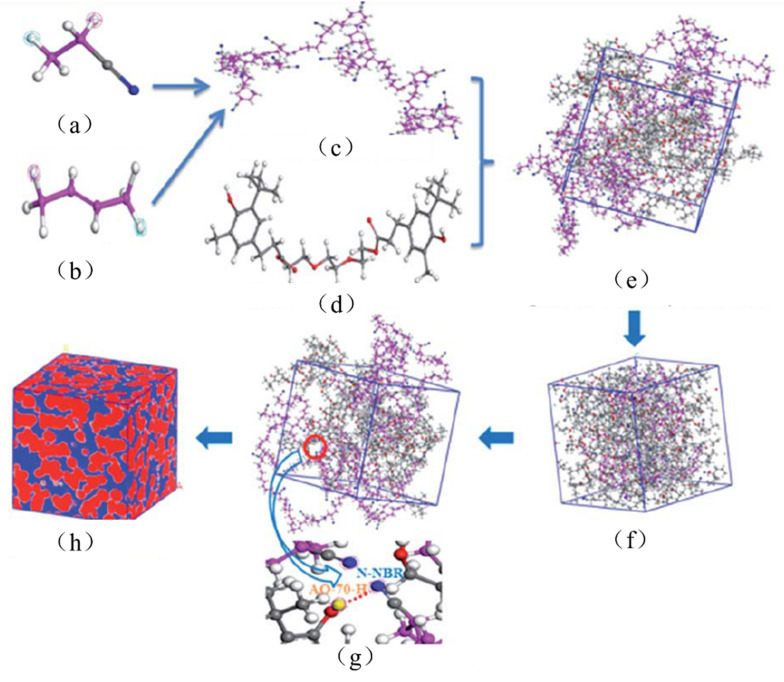
Models for molecular dynamics simulation of AO-70/NBR rubber composites (blue sphere represents N atom, red sphere represents O atom, grey sphere represents H atom, pink sphere and dark grey sphere connects with N atom represent C atom, and red dashed line represents H-bond): (**a**) A Acrylonitril repeating unit; (**b**) A Butadiene repeating unit; (**c**) NBR polymer chain; (**d**) AO-70 molecule; (**e**) 3D cubic cell with periodic boundary conditions; (**f**) Molecular dynamics simulation; (**g**) H-bonds in AO-70/NBR and (**h**) Free volume analysis [40].

**Figure 9:**
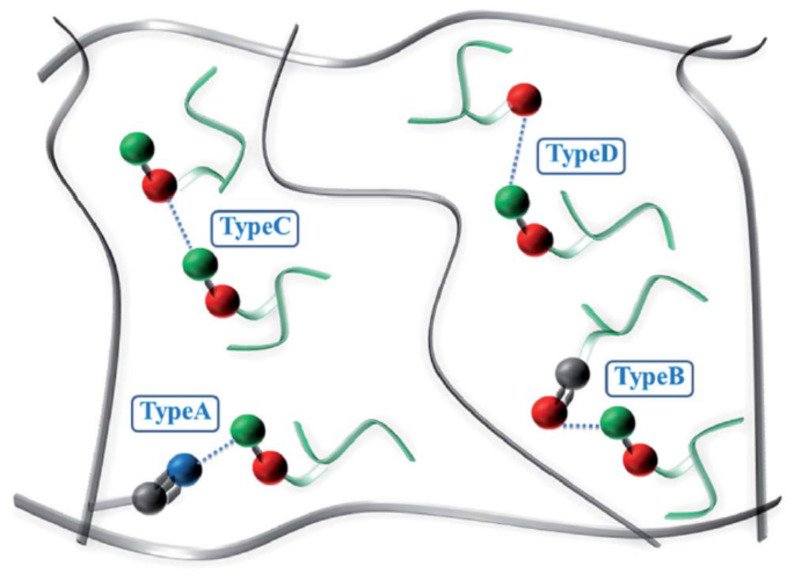
H-bond network in the AO-70/NBR rubber composites, (black thick lines, green short lines and blue dashed lines represent NBR polymer chains, AO-70 small molecules, and H-bonds, respectively. The black, blue, green and red balls represent carbon, nitrogen, hydrogen and oxygen atoms, respectively) [40].

**Figure 10:**
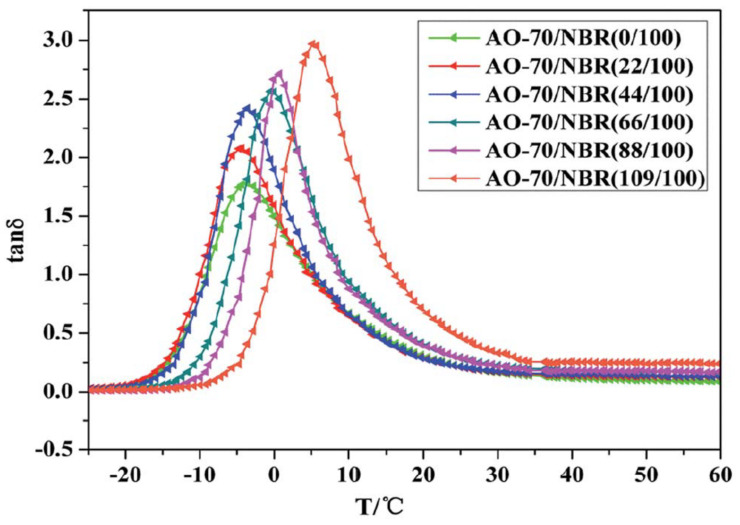
Temperature dependence of the damping loss factor (tan δ) of NBR and AO-70/NBR composites [40].

In [39], NR/NBR/AO-80 rubber composites with blend ratios of 75/25/10, 50/50/20 and 25/75/30 were further prepared for high-damping rubber isolation bearings. Morphology, damping properties and strain-induced orientation/crystallization of the materials were obtained using atomic force microscopy (AFM), DMTA analysis, polarized FTIR and tensile tests. DMTA analysis (temperature range of −100–100 °C, frequency of 10 Hz, heating rate of 3 °C/min) showed that the rubber materials had two damping peaks, in the low temperature range from −70 to −50 °C, respectively, and in the high temperature range from 15 to 25 °C, respectively. The high-temperature damping peak shifted to low temperatures with increase in NR composition due to the partial compatibility between NR and NBR/AO-80 phases. In addition, NR/NBR/AO-80 rubber composites had the effective damping temperatures of 51 and 32.3 °C with the damping loss factor greater than 0.1 and 0.3, respectively, which did not satisfy the requirements for the improvement of low-temperature resistance and the broadening of the effective damping temperature range of rubber materials, and they lay all above zero. Polarized FTIR showed that strain-induced orientation was produced in the strain range of 100–500%. Tensile test results illustrated that the tensile strength and the elongation at break of the rubber composites were all above 20 MPa and 650%, which satisfied the requirements of mechanical properties of high-damping rubber materials used for the production of high-damping rubber isolation bearings. In [40], models established for the molecular dynamics simulation of microstructure of NBR/AO-70 composites using Material Studio 7.0 software were constructed (Figure 8), and the results of dynamic mechanical analysis (DMA) were compared to investigate the relationship between the characteristics of hydrogen bond and the damping mechanism of NBR/AO-70 rubber composites. The computed results by molecular dynamics simulation (MDS) revealed that four types of hydrogen bonds (H-bonds), namely, type A (AO-70)–OH···NC–(NBR), type B (AO-70)–OH···O=C–(AO-70), type C (AO-70)–OH···OH–(AO-70), and type D (AO-70)–OH···O–C–(AO-70) were formed in the NBR/AO-70 composites after the addition of AO-70, as shown in Figure 9. DMA analysis showed that the addition of AO-70 significantly improved the damping properties of NBR/AO-70 rubber composites. When the blend ratio of NBR/AO-70 was 100/109, the rubber material had the largest hydrogen bonds and the optimal damping characteristics. The damping peak value was increased by 66.9% after adding AO-70, as shown in Figure 10.

### 4.4. ENR Composites

**Figure 11:**
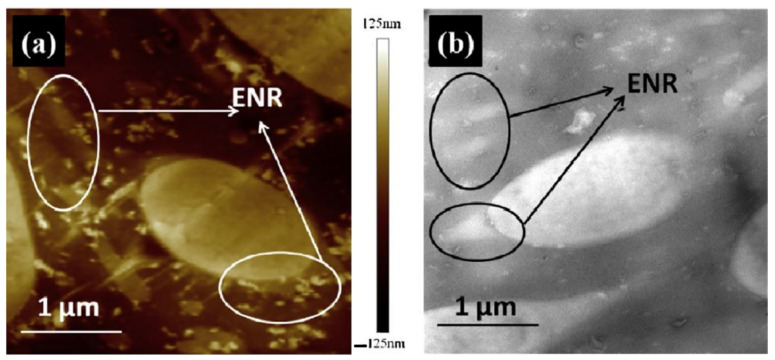
(**a**) AFM height image and (**b**) TEM micrograph of NR/NBR/ENR25 (70/30/15) composite [32].

**Figure 12:**
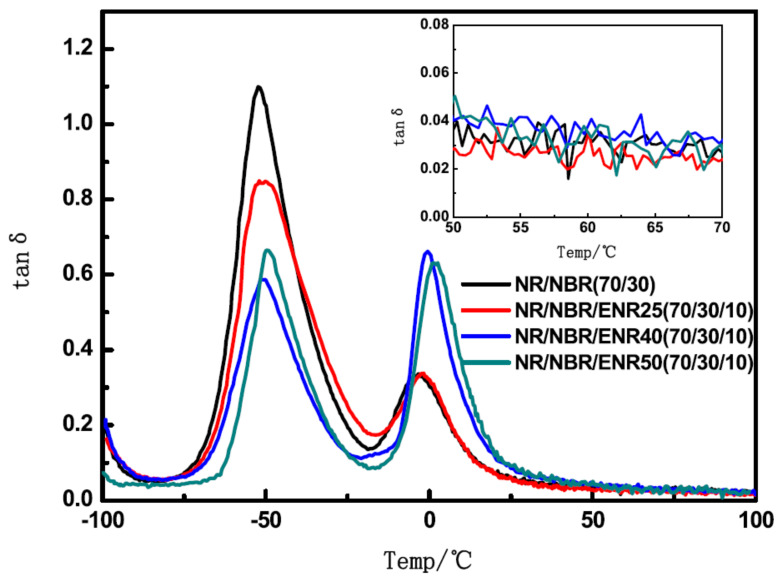
Temperature dependence of the damping loss factor (tan δ) of NR/NBR (70/30) and NR/NBR/ENR (70/30/10) composites with different epoxidation degrees of ENR [32].

Epoxidized Natural Rubber (ENR) is the product of the epoxidation of NR by peracetic acid [41]. ENR possesses outstanding mechanical properties of NR due to strain-induced crystallization [42]. Furthermore, the introduction of epoxy groups confers the ENR with different polarities and other properties such as oil resistance, low gas permeability, adhesion, anti-aging and high damping properties [43,44,45,46]. In [32], the effect of ENR with different epoxidation degrees on the damping performance of NR/NBR/ENR rubber composites, for which the NR/NBR/ENR composites with a blend ratio of 70/30/10, respectively, were prepared using ENR with epoxidation degrees of 25%, 40% and 50%, respectively. DMTA analysis (temperature range of −100–100 °C, frequency of 10 Hz, heating rate of 3 °C/min) showed that the addition of ENR reduced the aggregation of NBR phase and improved the compatibility between NR and NBR, as indicated by Figure 11. In the figure, the lighter and darker regions represent, respectively, the crosslinked NBR and NR phases. ENR is mainly dispersed in the NR matrix and gathers at the interface between NR and NBR, which develops the interfacial roughening and improves the interfacial adhesion in the immiscible NR/NBR rubber blends. The rubber composites exhibited two damping peaks at around −50 and 0 °C, respectively. With the increase in the epoxidation degree of ENR, the low-temperature damping peak of the rubber material decreased from 1.10 to 0.58, the high-temperature damping peak increased from 0.32 to 0.61, and the damping valley between two damping peaks decreased from 0.17 to 0.08, as illustrated in Figure 12. This can be explained by the fact that the polar ENR migrates from the NR matrix to the NBR phase because of the polarity difference between NR and NBR. The material had a low glass transition temperature (−52.03–49.96 °C) near the low-temperature damping peak. The damping loss factor fell below 0.1 when the temperature reached above 22 °C and it further decreased as the temperature rose. Therefore, the high-temperature environment and the temperature rise caused by the dissipated energy of the rubber material will lower the energy dissipation capacity of the rubber material.

**Figure 13:**
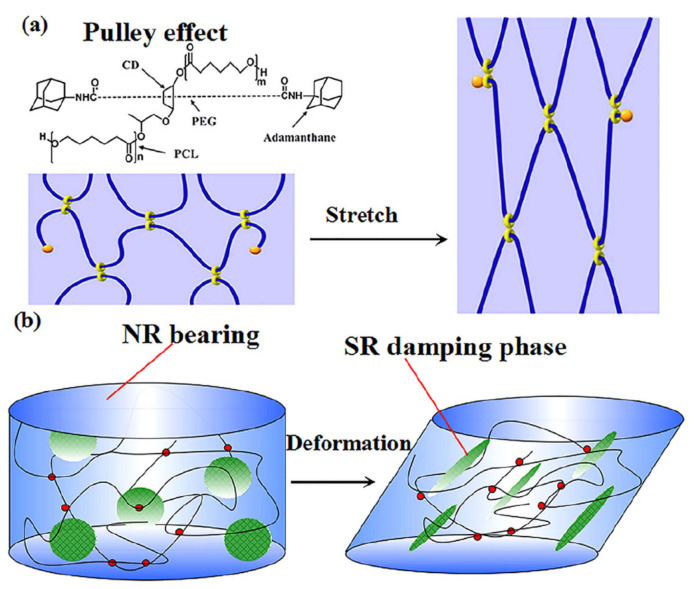
(**a**) Illustration of “Pulley effect” of slide-ring materials and (**b**) design concept of high-damping natural rubber/slide-ring composites [48].

**Figure 14:**
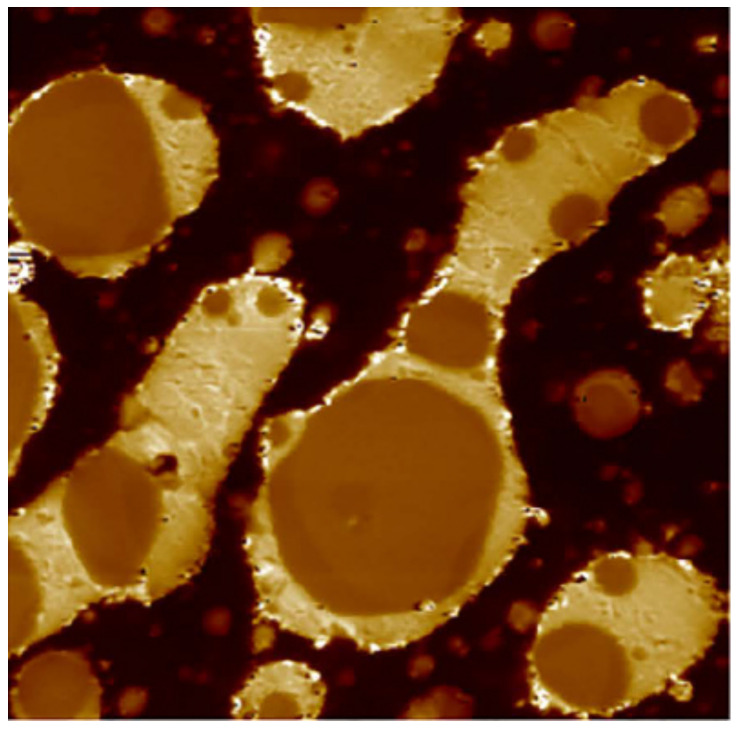
AFM images of NR/ENR/SR (70/30/40) rubber composites [48].

**Figure 15:**
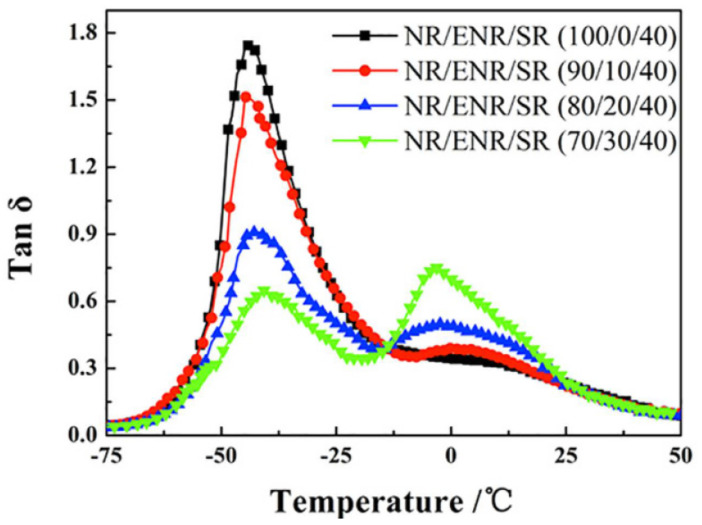
Temperature dependence of the damping loss factor (tan δ) of ternary NR/ENR/SR rubber composites [48].

In [47], molecular dynamics models of fixed-connection and slip-ring structures were constructed to investigate the effect of the slip-ring structure on the damping properties of rubber composites. Simulation results showed that the slip-ring structure had a slower mobility and a lower glass transition temperature compared with the fixed-connection structure when both structures had the same crosslink density. Moreover, the slippage of the molecular chain provided the slip-ring structure with high-damping characteristics, which facilitated the energy dissipation of the material under the action of external forces. In [48], NR/ENR/SR rubber materials with blend ratios of 90/10/40, 80/20/40 and 70/30/40, respectively, were prepared using a new polymer component, called the slip-ring “SR”, as a high-damping phase dispersed in rubber matrix. The design concept of high-damping rubber composites having the “pulley effect” is illustrated in Figure 13. The surface of the NR/ENR/SR (70/30/40) composites was imaged by AFM. As shown in Figure 14, an “elastic-coating” of ENR phase (the brightest region) on the SR phase can be observed, which improved the interfacial compatibility between SR and NR. DMTA analysis (temperature range of −100–100 °C, frequency of 10 Hz, deformation rate of 0.1%, heating rate of 3 °C/min) showed that when the composition ratio of ENR was higher than 20%, two damping peaks of the rubber material appeared, respectively, at around −44 and −2.5 °C, as shown in Figure 15. Compared to NR/SR two-phase rubber composites, NR/ENR/SR had a broad effective damping temperature range. The damping loss factor of the material increased with the increase in the composition ratio of ENR between 0.1 and 0.3 within the frequency range of 0.1–10 Hz at the strain of 5%, and within the strain range of 1–200% at the frequency of 1 Hz, respectively. The damping loss factor of the rubber material with the blend ratio of 70/30/40 was greater than 0.3 within the temperature range from −53 to 22 °C, namely, the effective damping temperature range reached 75 °C, which lay within the temperature range of 60–80 °C required for high-damping rubber materials. However, the increase in the composition ratio of ENR did not elevate the damping valley of the material and the damping loss factor decreased significantly when the temperature was greater than 25 °C.

Results of tensile tests showed that the tensile strength and the elongation at break of rubber materials with all these blend ratios fluctuate around 13.1 MPa and 781%, respectively, as tabulated in Table 2, which meets the standard requirements of high-damping rubber isolation bearings.

In [49], NBR/ENR50 (epoxidation degree of 50%) rubber composites with blend ratios of 70/30, 50/50 and 30/70 were prepared, and the damping and mechanical properties of NBR/ENR50 were obtained. DMA analysis showed that one damping peak appeared in the mixture of NBR and ENR50 due to the strong polar interaction between two polar rubbers NBR and ENR50. With the increase in the composition ratio of ENR50, the damping peak varied within the temperature range from −8 to 7.5 °C, and slightly shifted towards high temperatures. The maximum of the damping loss factor of NBR/ENR50 rubber blends with a blend ratio of 50/50 can reach as high as 1.61, while the material had an effective damping temperature of 38 °C (−13 to 25 °C), where the damping loss factor was greater than 0.3. In the temperature range from −10 to 6.6 °C, the glass transition temperature of NBR/ENR50 increased with the increase in the vibration frequency (0.1–100 Hz). In addition, when the blend ratio was 50/50, the tensile strength and the elongation at break of the material reached their maximums of 3.69 MPa and 484%, respectively, which do not meet the requirements of high-damping rubber isolation bearings (Chinese Standard JG/T-118-2018 [26]).

### 4.5. EPDM Composites

**Figure 16:**
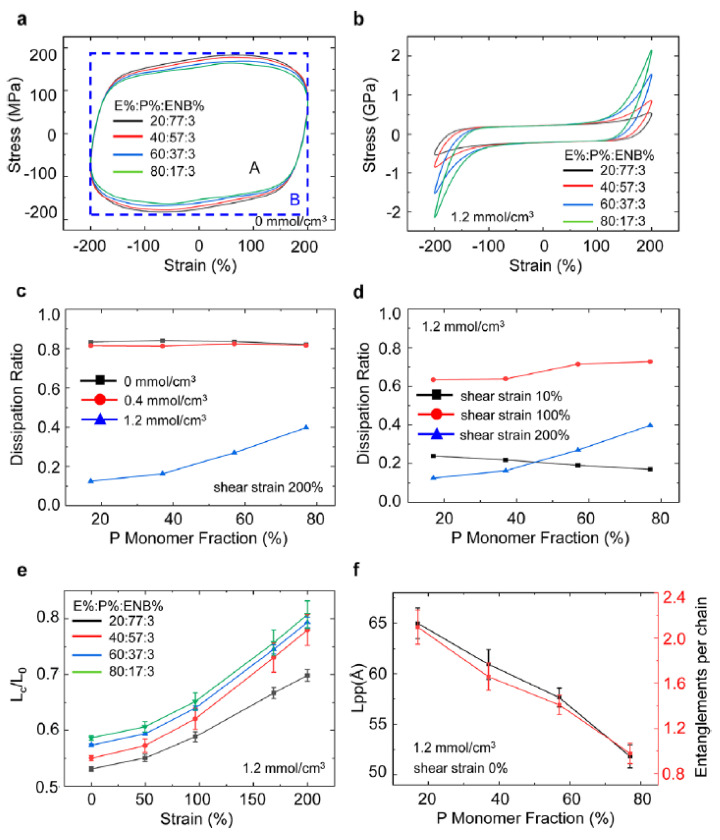
Influence of molecular design parameters on the EPDM dissipation ratio under large-strain deformation with a high frequency: (**a**) stress−strain curves of un-crosslinked EPDM with different ethylene (E), propylene (P), and ENB fractions; (**b**) stress−strain curves of crosslinked EPDM with different monomer fractions; (**c**) dissipation ratio of EPDM under different propylene fractions with varying crosslink densities at the shear strain of 200%; (**d**) dissipation ratio of highly crosslinked EPDM under different propylene fractions with varying shear strain amplitudes; (**e**) normalized mean distance between crosslinks of highly crosslinked EPDM under different propylene fractions with varying shear strain amplitudes; (**f**) primitive path length and entanglements per chain of highly crosslinked EPDM at the shear strain of 0% [54].

Ethylene-propylene-diene rubber (EPDM) is a highly saturated non-polar terpolymer composed of propylene, ethylene and a small amount of dicyclopentadiene. The molecular structure with saturated and stable backbone endows EPDM with excellent aging resistance, oxidation resistance and chemical resistance [50], and EPDM is able to maintain an outstanding flexibility and the resistance of compressive deformation at low temperatures. Thus, EPDM could provide a property of low-temperature resistance for the development of new high-damping rubber materials. In [51], the effects of ethylene content and crosslink density on the behaviours of tensile orientation and strain-induced crystallization of EPDM were investigated. It was found that the strain-induced crystallization occurred in EPDM, which contains 70% of ethylene content during the stretching process, and the tensile orientation and the strain-induced crystallinity first increased and then decreased with the increase in crosslink density. In [52], tensile and shear deformation tests were performed, respectively, on the neoprene (CR) and NBR/EPDM rubber composites with a blend ratio of 70/30. Results showed that both rubber materials had comparable mechanical properties under the shear strain of 200%. The maximum tensile strength and the elongation at break of NBR/EPDM blends could reach 14.6 MPa and 215%, respectively. Considering the low cost and short vulcanization period of NBR/EPDM blends, it could be an appropriate rubber material for the production of seismic isolation rubber bearings. However, NBR/EPDM rubber composites cannot be directly used alone as a rubber matrix material for industrial applications [53]. In [54], a series of amorphous EPDM polymer systems were established using all-atom molecular dynamics simulations to elucidate the effects of chemical composition and crosslink density on the linear and nonlinear viscoelasticity of EPDM elastomer in glass and glass-rubber transition regimes under different strain rates. The simulation results, as illustrated by Figure 16, revealed that increasing the crosslink density is the most effective way to decrease the dissipation ratio of EPDM under large shear deformation. This approach will provide a way for multiscale analysis, linking chemical composition and microstructure to the material performance.

**Figure 17:**
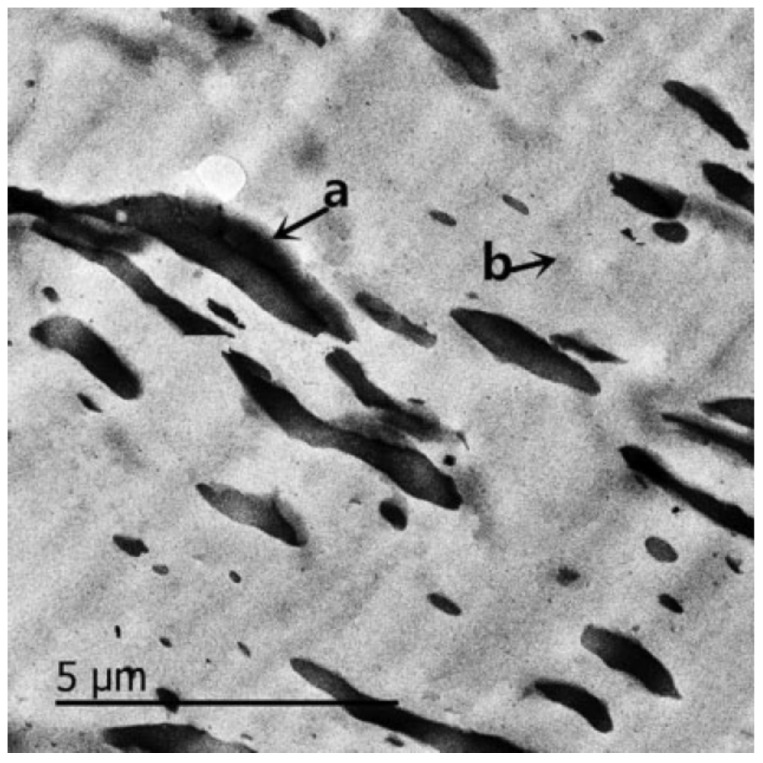
TEM images of EPDM/ENE50 (75/25) blend: (**a**) ENR50 phase, and; (**b**) EPDM phase [55].

**Figure 18:**
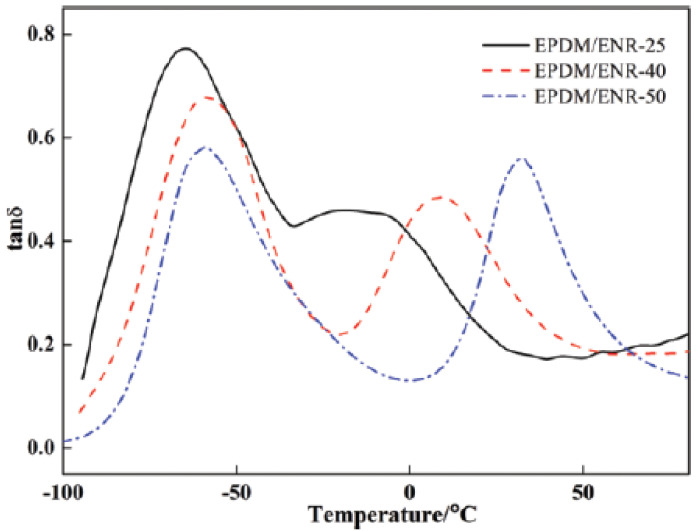
Temperature dependence of the damping loss factor (tan δ) for EPDM/ENRs (75/25) blends with ENRs of different epoxidation degree [55].

**Figure 19:**
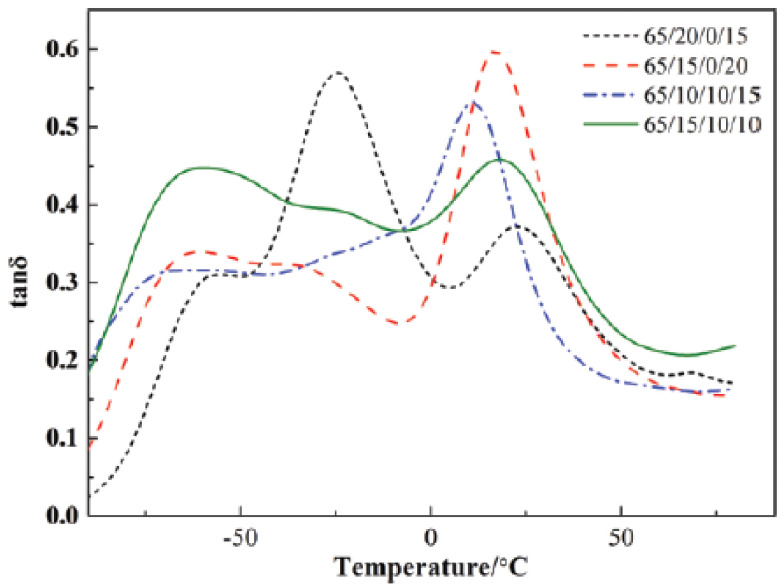
Temperature dependence of the damping loss factor (tan δ) for EPDM/ENR50/ENR40/ENR25 multicomponent blends [55].

**Figure 20:**
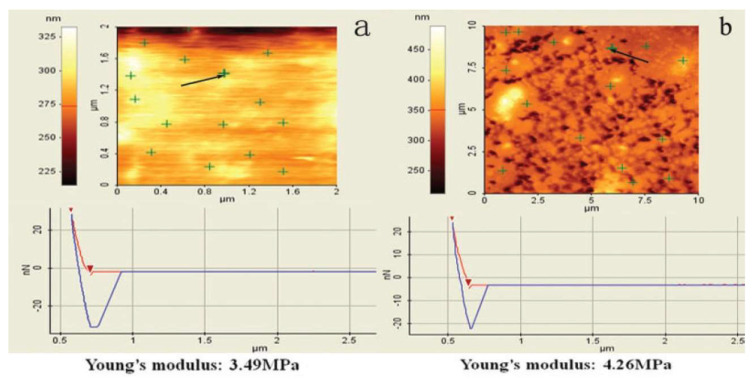
Young’s modulus calculation results of ENR50: (**a**) pure cured ENR50, and; (**b**) ENR50 in EPDM/ENR50 (75/25) blend [55].

**Figure 21:**
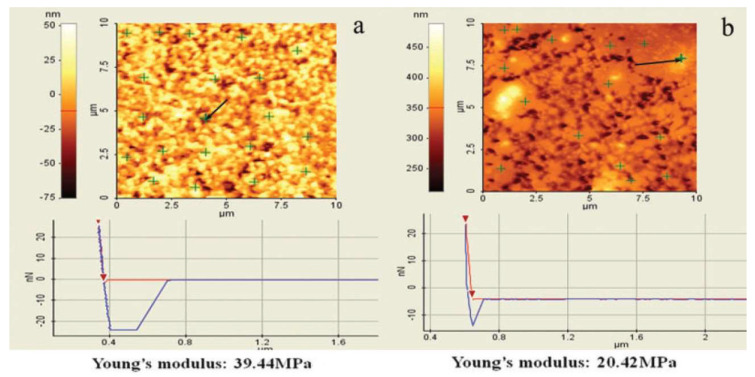
Young’s modulus calculation results of EPDM: (**a**) pure cured EPDM, and; (**b**) EPDM in EPDM/ENR50 (75/25) blend [55].

In [55], the effect of different epoxidation degrees of ENR (epoxidation degrees of 25%, 40% and 50%) on broadening the effective damping temperature range of ENR/EPDM rubber blends was studied. A “sea island” structure of the material was obtained, and illustrated by TEM image, as shown in Figure 17. ENR50 was dispersed as the “island” phase in the continuous EPDM phase as the “sea” phase. DMTA analysis (temperature range of −100–100 °C, frequency of 10 Hz, power of 5 N, heating rate of 4 °C/min) illustrated that the high-temperature damping peak of ENR/EPDM shifted towards high temperatures with the increase in epoxidation degree of ENR within the temperature range from −8.3 to 31.3 °C, as shown in Figure 18. Based on this observation, EPDM/ENR50/ENR40/ENR25 multi-component rubber composites with blend ratios of 65/10/10/15 and 65/15/10/10 were further prepared. It was found that EPDM/ENR50/ENR40/ENR25 rubber blends had a damping loss factor, which was greater than 0.3 over the temperature range of −80.2–40.3 °C with an effective damping temperature of 120.5 °C, as shown in Figure 19. Compared to the pure ENR50 and EPDM, the Young’s modulus of ENR50 in the blend increased from 3.49 MPa to 4.26 MPa, while the Young’s modulus of EPDM in the blend decreased from 38.39 MPa to 20.42 MPa, as shown in Figure 20 and Figure 21, respectively. The difference in the Young’s modulus of each phase between the pure one and the one in the blend reflects the variation in their crosslink extent before and after blending, which might be caused by the migration of vulcanizers and accelerators from EPDM phase to ENR50 phase.

In [50], petroleum resin (C94) was used to prepare EPDM/C94/NBR terpolymers with blend ratios of 70/30/10, 70/30/30 and 70/30/100, respectively, and the effect of C94 on their damping properties was investigated. DMTA analysis (temperature range of −80–150 °C, frequency of 11 Hz, strain amplitude of 3 μm, heating rate of 3 °C/min) showed that increasing the content of C94 enabled the damping peak of EPDM to move towards high temperatures within the temperature range from −40 to 40 °C. With the addition of NBR, the effective damping temperature range of EPDM/C94 with a damping loss factor greater than 0.3 was significantly broadened to 185 °C (−35–150 °C).

### 4.6. IIR, CIIR, and BIIR Composites

**Figure 22:**
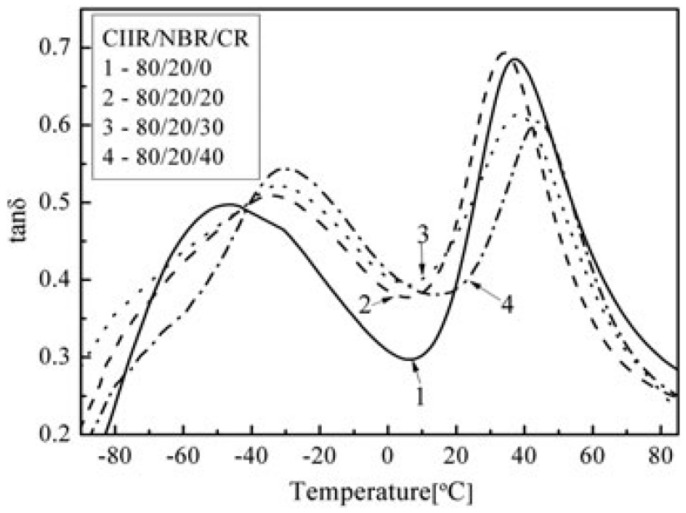
The temperature dependence of the damping loss factor for CIIR/NBR/CR ternary blends [57].

**Figure 23:**
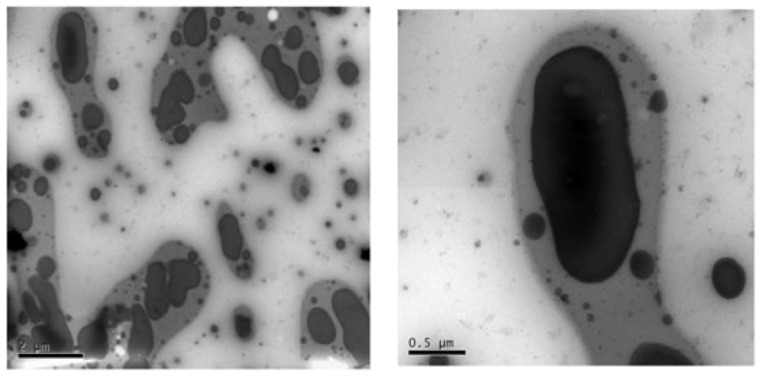
TEM images of CIIR/NBR/CR (80/20/30) blends [57].

**Figure 24:**
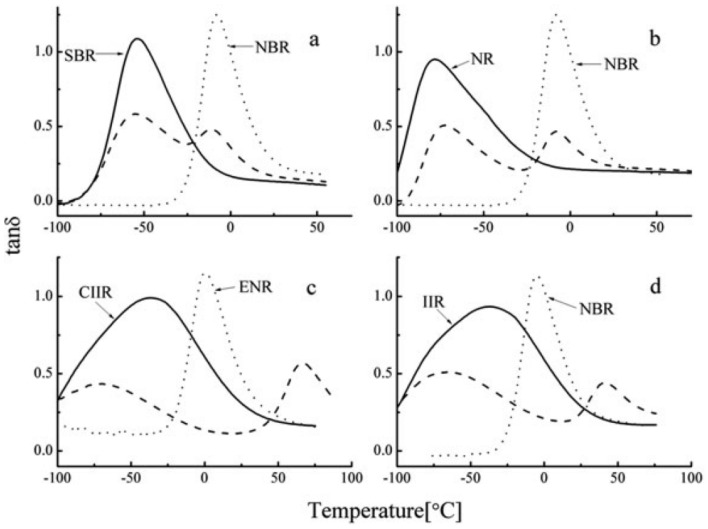
Temperature dependence of the damping loss factor (tan δ > 3) of other rubber blends such as (**a**) SBR/NBR; (**b**) NR/NBR; (**c**) CIIR/ENR and (**d**) IIR/NBR binary blends [57].

Butyl rubber (IIR) is a low unsaturated synthetic rubber copolymerized with isobutylene and a small amount of isoprene, which has excellent damping properties. In [56], NR and butyl rubber (IIR) were used as rubber matrix materials, isobutylene-isoprene block copolymer (IIBC) as compatibilizer, to prepare NR/IIR/IIBC rubber composites with blend ratios of 80/20/4, 80/20/8 and 80/20/12 for the production of high-damping rubber isolation bearings. DMTA analysis (temperature range of −80–50 °C, frequency of 10 Hz, strain amplitude of 0.3%, heating rate of 3 °C/min) showed that a damping peak appeared in the rubber composite with a blend ratio of 80/20/4. The material had an effective damping temperature of 42 °C (−63–−21 °C) where the damping loss factor (tan δ) was greater than 0.3, and the damping peak decreased with the increase in IIBC composition. Results of electronic tensile tests showed that the NR/IIR/IIBC rubber composites with a blend ratio of 80/20/4 had the highest static mechanical properties (tensile strength, Shore hardness, 100% and 200% modulus at constant elongation and elongation at break). The tensile strength and the elongation at break could reach about 21.6 MPa and 690%, respectively. Obtained results using the rubber processing analyzer (RPA) showed that the damping loss factor was greater than 0.1 in the frequency range of 0.2–5 Hz and in the strain range of 50–200%, while it became less than 0.1 in the strain range of 10–50%. However, the modified rubber materials of IIR, namely chlorinated butyl rubber (CIIR) or bromine butyl rubber (BIIR) are commonly used, instead of IIR, to prepare rubber damping materials due to drawbacks of slow vulcanization speed and difficulty in blending and co-vulcanization of IIR with other rubber materials [25,29]. In [57], CIIR/NBR/CR rubber composites with blend ratios of 80/20/20, 80/20/30, 80/20/40 were prepared, and their damping properties were obtained. DMA analysis (temperature range of −100–80 °C, frequency of 10 Hz, heating rate of 3 °C/min) showed that two damping peaks appeared, respectively, at around −33 and 40 °C. Furthermore, the addition of CR significantly increased the damping valley between two damping peaks, as shown in Figure 22. The effective damping temperature range (tan δ > 0.3) of CIIR/NBR/CR rubber composites was broadened to 161 °C (−86.4–74.6 °C) when the blend ratio was 80/20/30. This can be explained by the morphology of CIIR/NBR/CR blends obtained by TEM (Figure 23). The gray region between NBR (dark region) and CIIR (light region) phases is the phase of CR layer. This interfacial transition layer helps the slip of the interface region increase when the temperature is between the glass transition temperatures of CIIR and NBR. In addition, it was found that all rubber blends of CIIR/ENR, IIR/NBR, SBR/NBR and NR/NBR had two damping peaks, as shown in Figure 24, and their glass transition temperatures were (−70 °C, 66.6 °C), (−66.7 °C, 41.6 °C), (−54.2 °C, −12 °C) and (−71.4 °C, −6.3 °C), respectively. It can be judged from Figure 24 that the blends of one unsaturated rubber and one saturated rubber could form a broader damping platform compared with those of two saturated rubbers.

**Figure 25:**
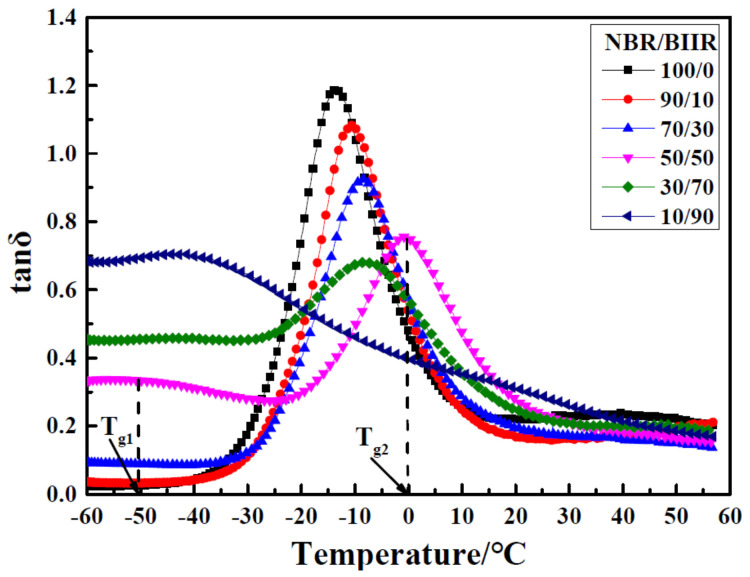
Damping loss factor versus temperature for NBR/BIIR with various blend ratios [31].

**Figure 26:**
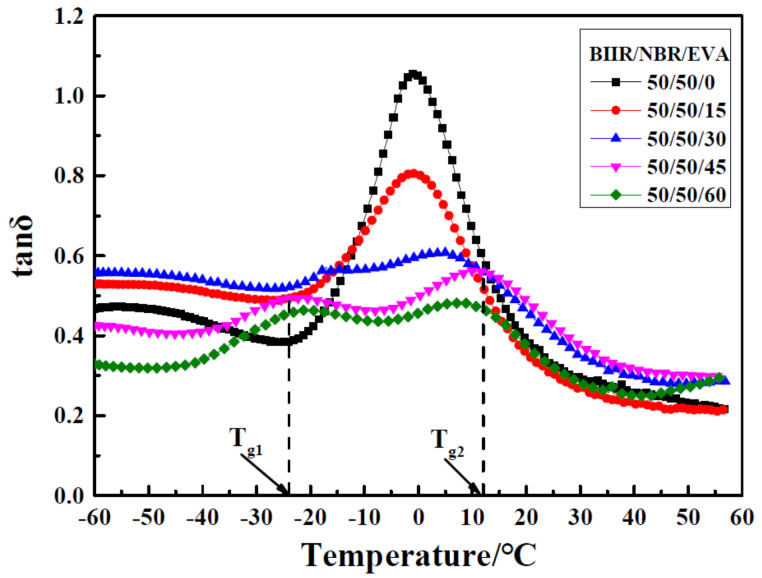
Damping loss factor versus temperature for NBR/BIIR/EVA with various blend ratios. [31].

In [31], bromine butyl rubber (BIIR) and NBR were used as rubber matrix materials, and ethylene-vinyl acetate copolymer (EVA) as the compatibilizer, to prepare NBR/BIIR and NBR/BIIR/EVA rubber composites with different blend ratios. DMA analysis showed that NBR/BIIR rubber blends had the optimal damping and mechanical properties when the blend ratio reached 50/50, and two damping peaks appeared at −50 and 0 °C, respectively, as shown in Figure 25. However, the effective damping temperature range (tan δ > 0.3) of NBR/BIIR was concentrated in the low temperature range below 0 °C, and the damping valley value of NBR/BIIR (50/50) was only 0.28. The NBR/BIIR/EVA rubber composites were prepared by adding EVA. Figure 26 implies that the effective damping temperature range of NBR/BIIR/EVA rubber composites was further expanded to 102 °C (−60–42 °C) when the blend ratio was 50/50/30, and the damping valley of NBR/BIIR/EVA (50/50/30) rubber composites was increased to 0.54 compared to NBR/BIIR (50/50) rubber composites.

**Figure 27:**
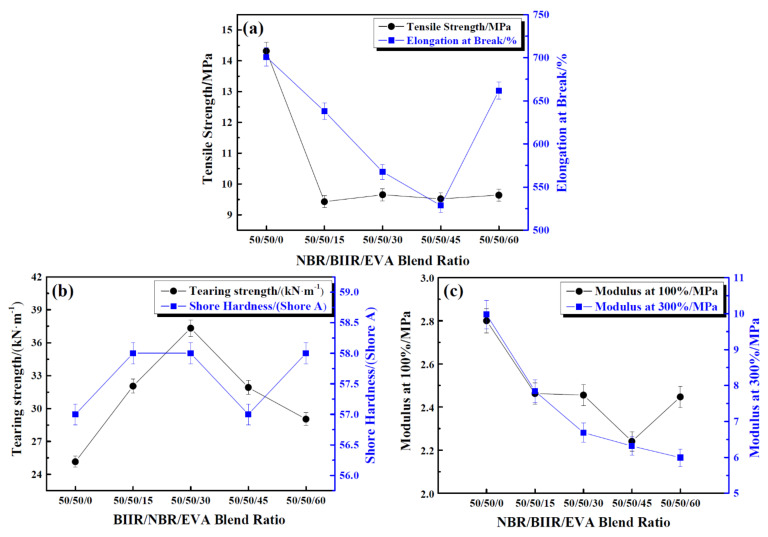
Mechanical properties of NBR/BIIR/EVA with various blend ratios: (**a**) Effect of tensile strength and elongation at break; (**b**) effect of tear strength and Shore hardness, and; (**c**) effects of 100% and 300% modulus [31].

Evolutions of mechanical properties of NBR/BIIR/EVA rubber composites with various blend ratios such as the tensile strength, the elongation at break, the tear strength, the Shore hardness, 100% and 300% modulus are illustrated in Figure 27. Comparing both the damping and mechanical properties of NBR/BIIR and NBR/BIIR/EVA, rubber composites indicated that the addition of EVA improved the compatibility between NBR and BIIR interfaces, broadened the effective damping temperature range and increased the damping valley of NBR/BIIR rubber blends, while it degraded the mechanical properties of NBR/BIIR rubber blends.

**Figure 28:**
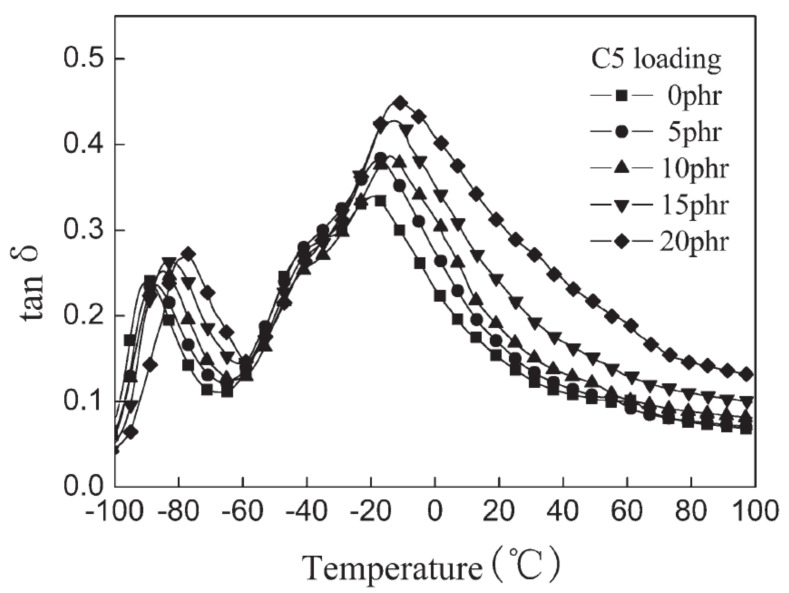
Temperature dependence of the damping loss factor (tan δ) for BIIR/BR/C5 vulcanizates with different loading of C5 resin [58].

**Figure 29:**
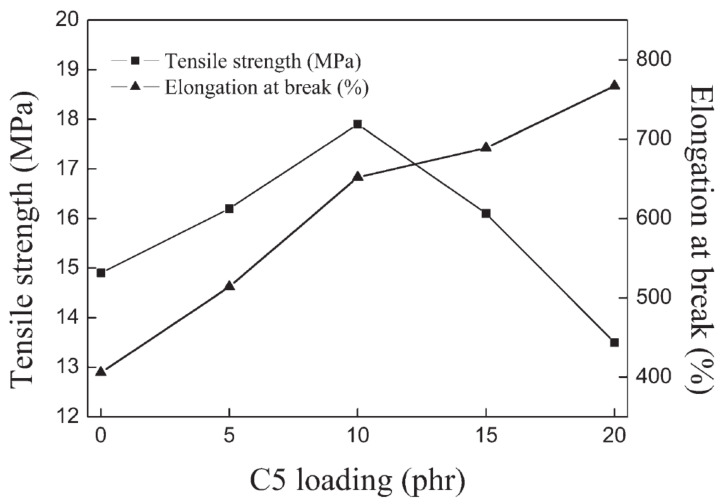
Tensile strength and elongation at break versus the loading of C5 resin for BIIR/BR/C5 vulcanizates [58].

In [58], rubber composites consisting of white-silica-filled vulcanizates of bromine butyl rubber (BIIR)/cis-1,4-polybutadiene rubbers (BR) with hydrogenated aromatic hydrocarbon (C5) petroleum resins were prepared, and the effect of C5 resin loading on the damping and mechanical properties of the material was studied. DMA analysis (temperature range of −100–100 °C, frequency of 10 Hz, static strain of 5%, dynamic strain of 0.5%, and heating rate of 2 °C/min) exhibited two damping peaks near −80 and 0 °C corresponding to the glass transition temperatures of BR and BIIR, respectively. With the increase in C5 resin content, the values of both damping peaks increase, and both damping peaks of rubber blends shifted significantly towards high temperatures, as shown in Figure 28. It can be seen from the figure that the effective damping temperature range of the rubber blends (tan δ > 0.3) was remarkably broadened from 34.1 to 54.2 °C with the increase in the C5 resin content from 5 phr to 20 phr, especially in the temperature range of the BIIR glass transition. However, the width of the damping peak in the temperature range of BR glass transition was slightly changed with the addition of the C5 resin due to the high cis synthetic and low hysteresis compared to the BIIR. In addition, the tensile strength reached its maximum of 18 MPa when the C5 resin content reached 10 phr, at which the elongation at break reached around 650%, as shown in Figure 29.

## 5. Vulcanization System

**Figure 30:**
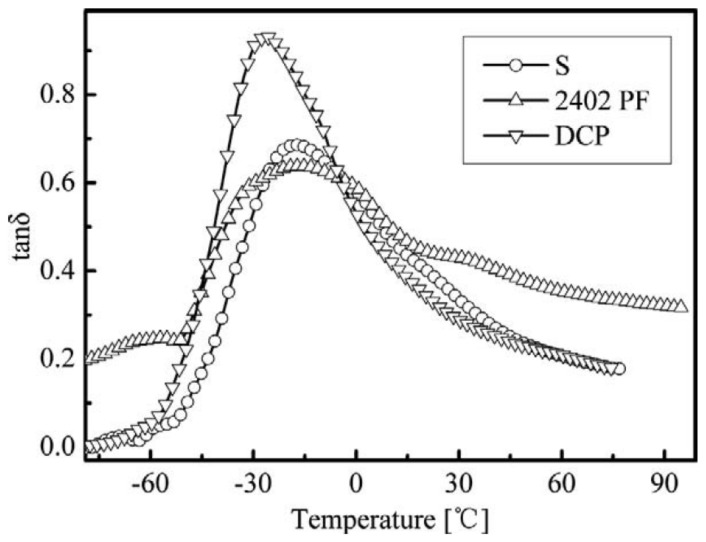
Evolution of the damping loss factor of ENR vulcanized by S, DCP and 2402 PF with respect to the temperature [59].

**Figure 31:**
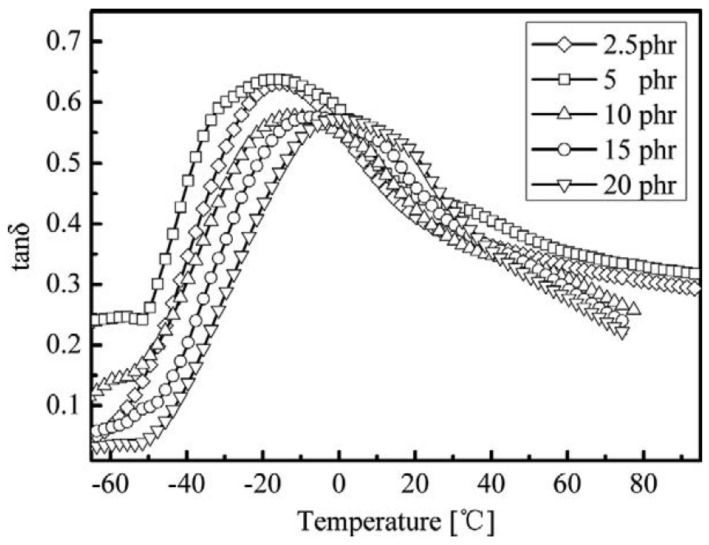
Evolution of the damping loss factor of ENR vulcanized by different content of 2402 PF with respect to the temperature [59].

Different vulcanizing agents and accelerators will affect the damping and mechanical properties of rubber composites. In [59], the effects of sulfur (S), peroxide (DCP) and phenolic resin (2402 PF) on broadening the damping temperature range of ENR were investigated. As shown in Figure 30, DMA analysis of ENR vulcanizates showed that the effective damping temperature range of ENR vulcanized by 2402 PF was broader than that of ENR using other vulcanization systems, due to the hydrogen bonds formed between ENR and phenolic resin (2402 PF). It was further found that ENR vulcanized by 5 phr of 2402 PF had the broadest damping temperature range of 147.6 °C (−47.6–100 °C), as shown in Figure 31.

**Figure 32:**
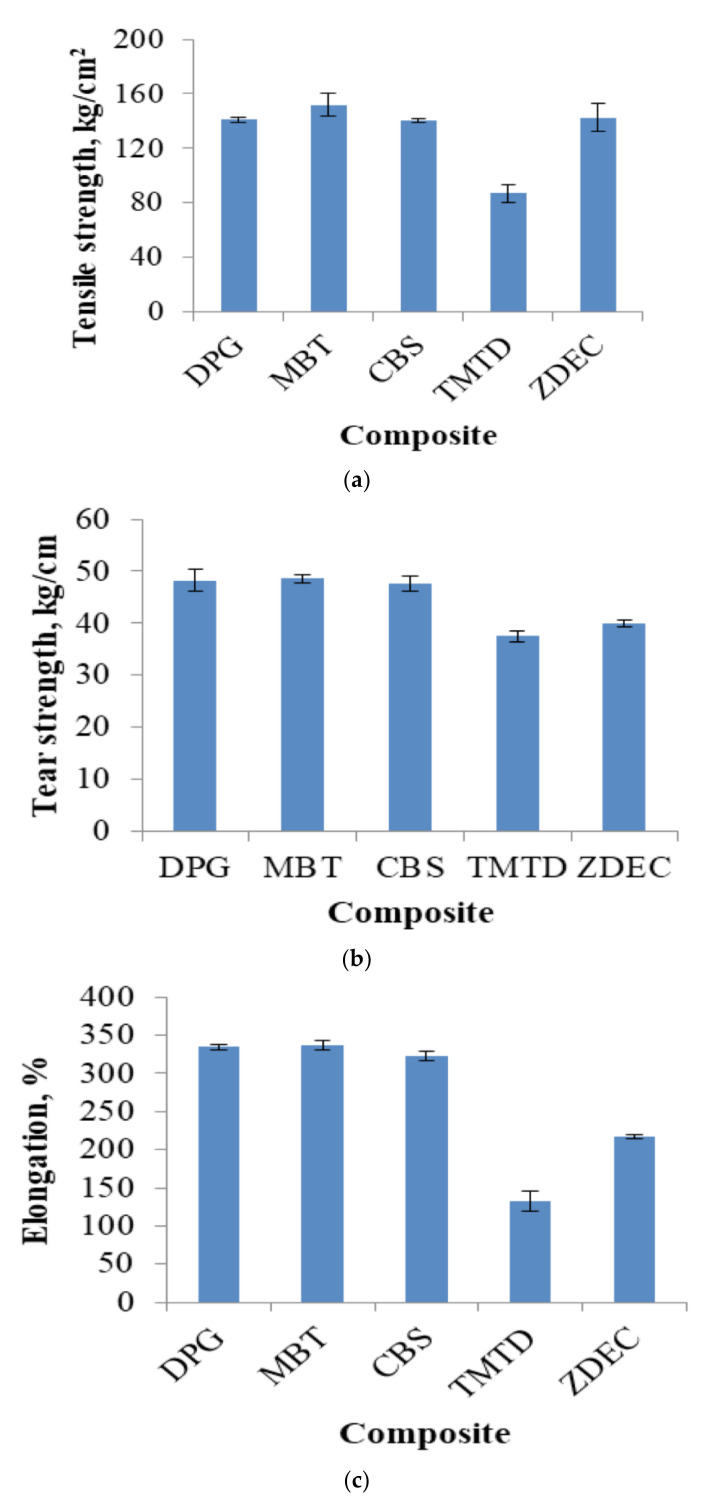
Mechanical properties of NBR/EPDM rubber composites: (**a**) tensile strength; (**b**) tear strength, and; (**c**) elongation [53].

In [53], the effects of different accelerators (DPG, MBT, CBS, TMTD, and ZDEC) on the vulcanization characteristics and the mechanical properties of montmorillonite-solubilized NBR/EPDM rubber composites were studied. Through a comparison of scorch time and optimal cure time of different accelerators, it was found that tetramethylthiuram disulfide (TMTD) was an ideal accelerator for the improvement of the vulcanization characteristics of the material. Through the comparison of tensile strength, tear strength and elongation of different accelerators, 2-mercaptobenzothiazole (MBT) was found to be an ideal accelerator for the improvement of mechanical properties of NBR/EPDM, as shown in Figure 32. It was additionally found that the damping loss factor decreased with the increase in the difference between the maximum and minimum torques of vulcanization.

In [60], vulcanization systems used to vulcanize butyl rubber (IIR) and halogenated butyl rubber (CIIR or BIIR) were described, and the effects of different vulcanization systems on the vulcanization characteristics and the mechanical properties of the butyl rubber and the halogenated butyl rubber vulcanizates were investigated. Compared to IIR, the vulcanization process of the halobutyl rubber is faster and the resulting vulcanizates have a higher crosslink density. Moreover, halobutyl rubbers are more likely to co-vulcanize with unsaturated rubbers due to the higher vulcanization rate. In addition, BIIR has a higher vulcanization rate and consumes less vulcanizing agent than CIIR does, due to the higher reactivity of carbon-bromine bonds than carbon-chlorine bonds [61].

## 6. Filler Reinforcement System

In rubber modification technology, carbon black is one of the most common fillers used for the reinforcement of rubber composites [62]. As a reinforcing agent for rubber materials, carbon black helps to improve the mechanical properties of the rubber and prolong its life span of service. In [63], the effect of carbon black on the damping properties and the tensile stress relaxation properties of EPDM was studied. The experimental results showed that the addition of carbon black reduced the damping peak of EPDM from 1.15 to 0.67, and shifted the glass transition temperature of the material towards low temperatures from −35 to −40 °C, while the damping loss factor of the material in the rubbery region above 0 °C was significantly increased to above 0.3. In addition, the results of stress relaxation tests showed that the relaxation modulus of carbon black filled EPDM exhibited a maximum variation of 2.5 MPa in the strain range from 1.5% to 10%.

The vulcanized NBR reinforced by carbon black has a high tensile strength. In [64], the effects of different carbon black fillers (N330, N880, white carbon black) and different ratios of white carbon black/carbon black (10%, 20%, 30% and 40%) on the mechanical properties of NBR were first studied. Second, the effect of temperature on the tensile strength and the elongation at break of filled NBR was investigated. Test results using a universal testing machine showed that carbon black N330 enabled NBR to possess better mechanical properties such as the tensile strength, the hardness, the elongation at break and the compression elastic modulus than the other types of carbon black. The tensile strength, the tear strength, the elongation at break and the thermal resistance were improved, while the compression elastic modulus was degraded with the increase in the composition ratio of white carbon black. However, the application of carbon black produces environmental pollutions [65], and the carbon black has the tendency for agglomeration within the rubber matrix due to the large particle size [66]. For a long time, the application of nanofillers in rubber composites has became an effective way to improve the mechanical properties of rubber composites. A series of nanofillers have been used to reinforce rubber composites successfully, such as natural fibers [67], carbon nanotube (CNT) [68,69,70], nano clay [71,72,73], nano silica [74,75,76], nano titania [77,78,79], nano calcium carbonate [80,81,82].

**Figure 33:**
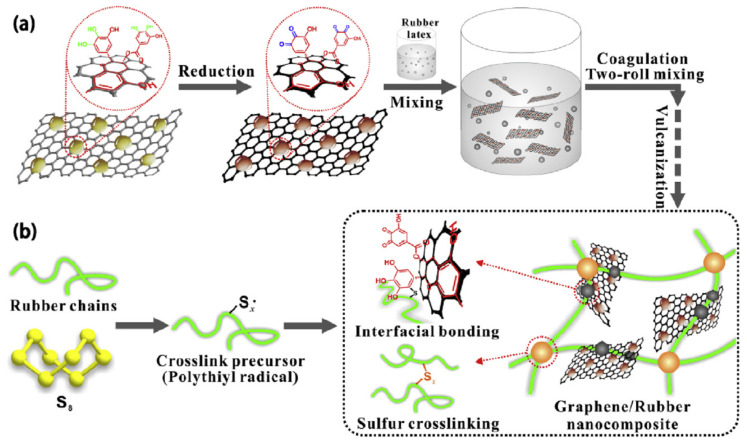
Schematic representations of the fabrication process of graphene/rubber nanocomposites and the formation mechanism of covalent interface between graphene and rubber matrix: (**a**) fabrication of graphene/rubber nanocomposites and (**b**) formation mechanism of covalent interface between graphene and rubber matrix [89].

In recent years, the development of rubber nanocomposites using graphene (GE) or graphene oxide (GO) as new nano-reinforced fillers has received extensive attention from researchers. Compared to other nanofillers, adding a small amount of GE or GO into rubber materials could achieve superior material properties such as damping and mechanical properties, and electrical and thermal conductivities; thus, GE or GO are considered as ideal multifunctional fillers for rubbers [83,84]. At present, the melt mixing and solution methods are the most widely used methods to prepare polymer nanocomposites [85]. Figure 33a illustrates a flow chart of fabricating graphene/rubber nanocomposites using latex mixing method followed by coagulation and vulcanization of the material. During the process, the sulfur crosslinking and the covalent interfacial bonding are, respectively, formed between molecular chain segments of the rubber, and between graphene sheets and rubber chain segments, as shown in Figure 33b. Fabricating rubber/graphene nanocomposites with excellent vulcanization, damping and mechanical properties requires the addressing of several issues that are focused on: (i) the homogeneous dispersion of graphene in rubber matrix; (ii) the impact mechanism of the graphene or graphene oxide on the vulcanization kinetics of rubber nanocomposites; (iii) designing a compact filler network in the rubber matrix, and; (iv) engineering a strong interfacial interaction between graphene and rubber matrix [86,87,88,89,90,91].

**Figure 34:**
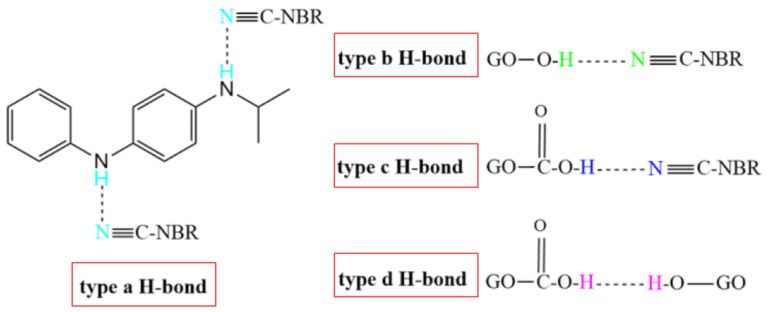
Four types of hydrogen bonds formed in NBR composites [96].

The special lamellar structure of graphene (GE) and the oxygen-containing functional groups on the surface of graphene oxide (GO) greatly affect the vulcanization, damping and mechanical properties of rubber materials [92]. Moreover, studies have found that GE and GO could act as accelerators and vulcanizers, respectively, in the vulcanization process of the rubber [93]. In [94], EPDM/NBR/GE/GO rubber nanocomposites using direct mixing method were prepared and the effect of GE/GO nanofillers on vulcanization, damping and mechanical properties of EPDM/NBR was investigated. The vulcanization test results showed that GE/GO nanofillers increased the maximum torque of vulcanization to 69 N·m and reduced the scorch time to 2 min, which increased the vulcanization rate. DMTA analysis showed that the peak value of the damping loss factor was decreased by 12% after the addition of GE/GO nanofillers. The results of tensile and rebound tests showed that the GE/GO nanofillers increased the tensile strength, 100% and 200% modulus of the rubber composites. With the increase in the composition ratio of nanofillers, the elongation at break and the rebound properties of rubber composites increase. In [95], the reinforcement effects of graphene on material properties such as the vulcanization rate, the tensile strength, the Young’s modulus and the elongation at break of NBR and EPDM rubbers were compared, as shown in Table 3. It was shown that the graphene has a significant reinforcement effect on both NBR and EPDM rubbers. A better reinforcement effect of the graphene on the polar NBR matrix than the non-polar EPDM matrix was observed due to strong hydrogen bonds (H-bonds) formed between the graphene and the polar NBR matrix. Four types of H-bonds (type a, b, c, and d), as shown in Figure 34, are formed in the NBR composites when GO is added, which improves the damping property of rubber matrixes [96]. A type a H-bond (Figure 34) is formed between -CN groups of NBR molecular chains and -NH- groups of small molecules 4010NA, which is an antioxidant grafted onto GO, expressed as (4010NA)-NH···NC-(NBR). Type b, c, and d H-bonds include H-bonds between -CN groups of NBR molecular chains and -OH groups of GO, expressed as (GO)-OH···NC-(NBR), H-bonds between -CN groups of NBR molecular chains and -COOH groups of GO, expressed as (GO)-COOH···NC-(NBR), and H-bonds between -OH groups of GO and -C-O-C- groups of GO, expressed as (GO)-O···HO-(GO).

**Figure 35:**
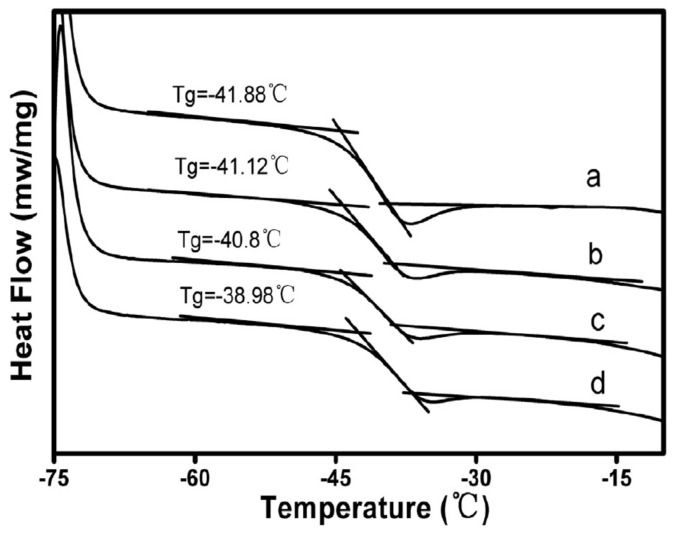
Glass transition temperature of (**a**) ENR; (**b**) SENR/GO-0.3; (**c**) SENR/GO-0.5, and; (**d**) SENR/GO-0.7 [97].

**Figure 36:**
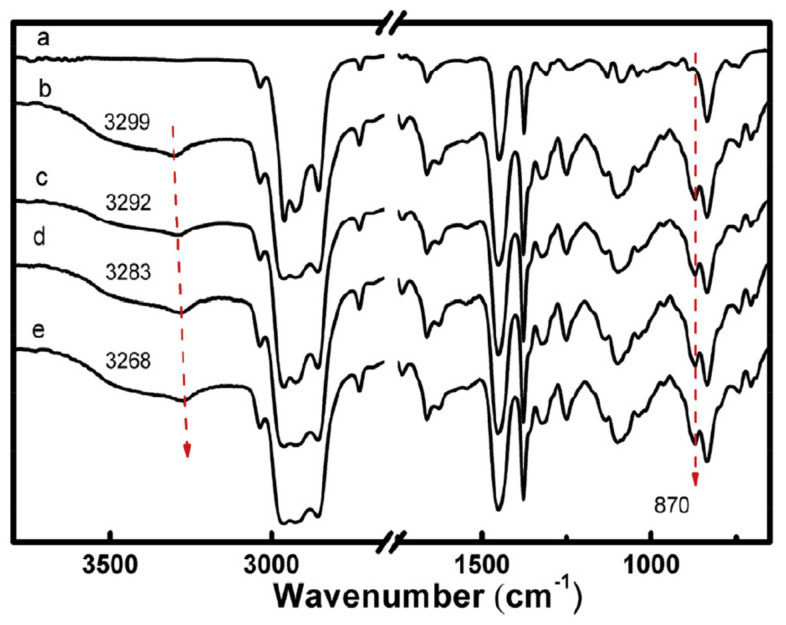
Normalized FTIR spectra of (**a**) NR; (**b**) ENR; (**c**) SENR/GO-0.3; (**d**) SENR/GO-0.5, and; (**e**) SENR/GO-0.7 [97].

**Figure 37:**
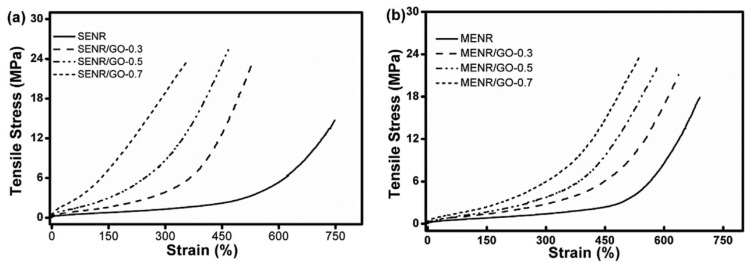
Representative stress-strain behavior for: (**a**) SENR/GO, and; (**b**) MENR/GO nanocomposites [97].

**Figure 38:**
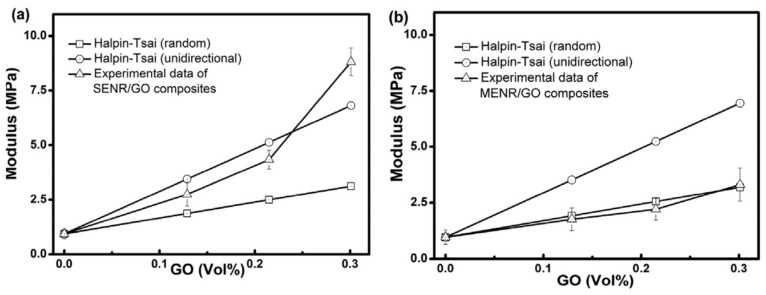
Experimental data of Young’s modulus (M200) and HalpineTsai theoretical model fitting for: (**a**) SENR/GO nanocomposites and (**b**) MENR/GO nanocomposites [97].

In [97], ENR/GO rubber nanocomposites using latex mixing method were prepared and the reinforcement effect of GO nanofillers on ENR was studied. Results of FTIR measurements, tensile tests and simultaneous thermo-analysis showed that, by adding 0.7 wt.% of GO into ENR, the glass transition temperature of ENR was increased from −41.88 °C to −38.98 °C, as shown in Figure 35, and the tensile strength of ENR was increased by 87%, and 200% modulus of ENR was increased to 8.7 times of that of ENR. The red-shift arrows in the FTIR measurements, as shown in Figure 36, indicated that new hydrogen groups were formed between epoxy and hydroxyl groups of ENR molecular chain and oxygenous groups (hydroxyls, carboxyls and epoxides) of GO sheets, which enhanced the interfacial interaction between the GO and ENR molecules, thereby improving the tensile strength and 200% modulus of ENR, as illustrated by Figure 37 and Figure 38. Note that SENR and MENR are abbreviations that represent the crosslinked ENR/GO nanocomposites vulcanized for 2 h at 100 °C and under a pressure of 15 MPa at 145 °C, respectively.

## 7. Constitutive Models of High-Damping Rubber Materials

Studying the thermodynamic constitutive behavior mechanism of high-damping rubber materials is an important means to investigate the evolution mechanism of mechanical properties of high-damping rubber materials. According to different theoretical frameworks, the development of constitutive models describing the hyperelastic and viscoelastic behaviors of rubber materials are mainly based on two approaches: phenomenological models within the continuum mechanics framework [98,99,100,101,102,103,104,105,106] and molecular network models based on statistical mechanics [107,108,109,110,111]. Up to now, some valuable constitutive models for carbon black filled rubber materials have been proposed. In [27,105,112], a set of generalized thermodynamic constitutive models for carbon black filled rubber materials have been proposed based on a rheological model combining the spring element, the Maxwell element and the friction element. Considering the rate dependence and the temperature dependence of viscosity of the rubber material, the model assumed the internal stress produced in the material as superimposition of equilibrium stress and overstress. The elastic effect of the equilibrium stress is described by the entropy elasticity combined with the modified Mooney–Rivlin model, while the viscous effect of the equilibrium stress is described by the friction element. In addition, the overstress generated by the Maxwell element expresses the rate dependence and temperature dependence of the material. However, the constitutive model proposed in this work did not consider Mullins effect, and the material functions used in the model do not conform to the relevant laws of polymer chemistry. In [113], uniaxial tensile tests on carbon black filled natural rubber materials at different temperatures were conducted. Based on the experimental results, an Arruda–Boyce model (eight-chain model) containing explicit temperature-dependent parameters was proposed. The comparison between the experimental and the numerical results showed that the model was able to estimate well the hyperelastic behavior of the rubber material in the strain range of 0–150% within the temperature range of 20–110 °C. On this basis, in [114], an exponential correction term was introduced to the Arruda–Boyce model and a modified Arruda–Boyce model was proposed to further consider the interaction effect between different components in the material. By comparing the ability of different models of the rubber material (Neo–Hookean [99], Mooney–Rivlin [98,100], Ogden [101], and Yeoh [103] models) in the prediction of experimental results, it showed that the proposed modified Arruda–Boyce model could more accurately estimate the static mechanical properties of carbon black filled natural rubber materials. However, at present, few research works have been reported on the temperature dependence of large deformation of graphene/graphene oxide filled high-damping rubber materials. The thermodynamic constitutive behavior mechanism of graphene/graphene oxide nano-reinforced high-damping rubber materials needs further investigations.

## 8. Analysis and Discussions

It can be seen from the aforementioned works, that the problems in using natural rubber (NR) as one of the components in the rubber matrix, sulfur vulcanization and carbon black filler-reinforcement systems to prepare high-damping rubber materials include: (i) NR can provide excellent mechanical properties for high-damping rubber materials, but the internal friction between NR molecular chains is very weak due to the flexibility and non-polarity of NR macromolecular chain segments, which causes a very low damping performance of NR, thereby hindering the improvement of damping properties of high-damping rubber materials. Moreover, the breakage of macromolecular chains and the formation of oxygen-containing groups will occur in practical applications of NR because of unsaturated double bonds and active allyl hydrogens in NR, which seriously degrade physical and mechanical properties of NR; (ii) sulfur vulcanized EPDM or IIR with a high saturation degree have very low vulcanization efficiency, which causes low mechanical properties of EPDM or IIR vulcanizates. Phenolic resin vulcanization can broaden the effective damping temperature range of ENR, but the efficiency of using phenolic resin to vulcanize ENR is very low, and it needs to be used together with the active agent of chloride to accelerate the vulcanization process of rubber, thereby increasing the vulcanization cost, and; (iii) large consumption of carbon black as reinforcement fillers, high-energy consumption of its preparation and hysteretic heat generation under dynamic loads will reduce the damping properties of high-damping rubber materials in the high temperature range and accelerate the thermal-oxidative aging of rubbers. Therefore, research on the preparation of high-performance high-damping rubber materials should be focused on the selection of other rubber materials to replace NR, the use of a suitable vulcanization system and further investigation of the reinforcement effect of graphene and graphene oxide on high-damping rubber materials.

On a microscopic scale, since it lacks an in-depth thermodynamic analysis of the compatibility between different rubber phases, using rubber blending technology to form homogenous, continuous and compatible rubber copolymers is always a problem that needs to be solved in the rubber blending process. In the vulcanization process, the type and dosage of vulcanizing agents, accelerators and activators are important factors to improve the vulcanization efficiency, to ensure the vulcanization safety, to control the degree of cross-linking, and to improve the processing performance and physical properties of rubber materials. Selecting appropriate vulcanizing agents, accelerators and activators, and balancing the chemical activity among these three are the key issues to be solved in the rubber vulcanization process. During the rubber blending, the vulcanizing agent and the accelerator migrate from the non-polar rubber phase to the polar rubber phase, which makes the non-polar rubber phase under-crosslinked, and the polar rubber phase over-crosslinked after vulcanization. This will cause the damping peaks in the low temperature and high temperature range to shift, respectively, towards low temperatures and high temperatures, which further expands the effective damping temperature range of rubber materials. Thus, it is necessary to further study the influence of vulcanization characteristics of high-damping rubber materials on their damping properties. Meanwhile, it lacks an in-depth understanding of the “sea island” effect of the damping loss factor of rubber materials, which hinders the synthesis of high-damping rubber materials with high and flat damping loss factor in a broad effective damping temperature range. Therefore, analyzing the generation mechanism of the “sea island” effect is a key issue to reveal the evolution mechanism of damping properties of high-damping rubber materials. This would provide a theoretical basis for broadening the effective damping temperature range, increasing the damping loss factor and weakening its temperature dependence of rubber materials. In addition, the interaction between fillers or between fillers and the rubber matrix will destroy the internal microstructure of rubber molecules, resulting in the Payne and Mullins effects of the rubber under dynamic strains. Thus, the analysis of motions of internal rubber phases and the impact of their interactions on the thermodynamic behavior of rubber materials is the key to revealing the evolution mechanism of mechanical properties of rubber materials. This will ensure rubber materials to be possessed of high mechanical properties when their damping properties are improved, and provide a basis for the improvement of their overall performance and maintaining their performance stability.

## 9. Conclusions and Perspectives

The performance of high-damping rubber materials largely determines the isolation performance of high-damping rubber isolation bearings. The damping and mechanical properties of high-damping rubber materials not only depend on the selection of rubber matrix materials, but also on the use of vulcanization and filler-reinforced systems. Furthermore, it is very urgent and necessary to develop high-performance high-damping rubber materials with a broad effective damping temperature range, high damping loss factor and weak temperature dependence to ensure the safety of seismic isolation of engineering structures. This paper first reviews the research progress in the development of high-damping rubber materials using nitrile butadiene rubber (NBR), epoxidized natural rubber (ENR), ethylene propylene diene rubber (EPDM), butyl rubber (IIR), chlorinated butyl rubber (CIIR), and bromine butyl rubber (BIIR) as rubber matrix materials. This is followed by the review of vulcanization and filler reinforcement systems that have been proposed for the improvement of damping and mechanical properties of high-damping rubber materials. Finally, it further reviewed constitutive models describing the hyperelasticity and viscoelasticity of rubber materials. In view of this focus, four key issues were highlighted: (i) the development of a suitable vulcanization system; (ii) the analysis of generation mechanism of “sea island” effect existing in the temperature dependence of the damping loss factor; (iii) the exploration of enhancement effect of graphene and graphene oxide fillers on high-damping rubber materials, and; (iv) the investigation of the influence of motions of inner rubber phases and their interactions on the thermodynamic behavior of rubber materials. Research in this field could provide new materials for the advancement of seismic isolation technology of engineering structures.

## Figures and Tables

**Table 1:** Mechanical properties of NBR/AO-80 nanocomposites [38].

Properties	Loadings of AO-80				
	0 phr	20 phr	40 phr	60 phr	100 phr
Hardness (Shore A)	59	57	63	75	88
Tensile strength (MPa)	3.1	10.9	16.0	17.3	15.0
Elongation at break (%)	373	536	608	703	653
Permanent set (%)	4	4	6	6	8

**Table 2:** Tensile properties of NR/ENR/SR rubber composites [48].

Test	NR/ENR/SR (90/10/40)	NR/ENR/SR (80/20/40)	NR/ENR/SR (70/30/40)
Tensile Modulus at 100% strain (MPa)	0.39 ± 0.03	0.41 ± 0.03	0.38 ± 0.03
Tensile Modulus at 300% strain (MPa)	0.96 ± 0.05	1.00 ± 0.05	0.95 ± 0.07
Tensile strength (MPa)	13.2 ± 0.37	13.3 ± 0.33	12.9 ± 0.35
Elongation at break (%)	782 ± 5.23	788 ± 5.46	773 ± 5.44
Hardness	29 ± 0.48	30 ± 0.39	31 ± 0.40

**Table 3:** Mechanical properties of NBR, EPDM and their nanocomposites [95].

Sample	Tensile Strength (MPa)	Young’s Modulus (MPa)	Elongation at Break (%)
NBR	4	3.9	337.0
NBR-GO-0.1	7.7 (92.5%)	13.2 (238.5%)	567.0 (68.2%)
NBR-GO-0.5	5.6 (40%)	16.8 (330.8%)	454.0 (34.7%)
NBR-GO-1	6.1 (52.5%)	19.7 (405.1%)	454.0 (34.7%)
EPDM	1.9	9.9	158.0
EPDM-GO-0.1	2.1 (10.5%)	15.5 (56.6%)	159.0 (0.6%)
EPDM-GO-0.5	2.2 (15.8%)	18.9 (90.9%)	175.0 (10.8%)
EPDM-GO-1	2.5 (31.6%)	23.3 (135.4%)	163.0 (3.2%)

## Data Availability

Not applicable.
